# Effect of membrane fusion protein AdeT1 on the antimicrobial resistance of *Escherichia coli*

**DOI:** 10.1038/s41598-020-77339-w

**Published:** 2020-11-24

**Authors:** Victoria L. Barlow, Shu-Jung Lai, Chia-Yu Chen, Cheng-Han Tsai, Shih-Hsiung Wu, Yu-Hsuan Tsai

**Affiliations:** 1 School of Chemistry, Cardiff University, Cardiff, UK; 2 Graduate Institute of Biomedical Sciences, China Medical University, Taichung, Taiwan; 3 Research Center for Cancer Biology, China Medical University, Taichung, Taiwan; 4 Institute of Biological Chemistry, Academia Sinica, Taipei, Taiwan

**Keywords:** Proteins, Proteins, Proteins, Proteins, Antimicrobial resistance

## Abstract

*Acinetobacter baumannii* is a prevalent pathogen that can rapidly acquire resistance to antibiotics. Indeed, multidrug-resistant *A. baumannii* is a major cause of hospital-acquired infections and has been recognised by the World Health Organization as one of the most threatening bacteria to our society. Resistance-nodulation-division (RND) type multidrug efflux pumps have been demonstrated to convey antibiotic resistance to a wide range of pathogens and are the primary resistance mechanism employed by *A. baumannii*. A component of an RND pump in *A. baumannii,* AdeT1, was previously demonstrated to enhance the antimicrobial resistance of *Escherichia coli*. Here, we report the results of experiments which demonstrate that wild-type AdeT1 does not confer antimicrobial resistance in *E. coli*, highlighting the importance of verifying protein production when determining minimum inhibitory concentrations (MICs) especially by broth dilution. Nevertheless, using an agar-based MIC assay, we found that propionylation of Lys280 on AdeT1 renders *E. coli* cells more resistant to erythromycin.

## Introduction

*Acinetobacter baumannii* is a Gram-negative bacterium and opportunistic human pathogen which has become a prevalent source of hospital-acquired infections^1^. Particularly, in intensive care units, *A. baumannii* accounts for 10% of infections worldwide^2^. Moreover, increasing rates of *A. baumannii* drug resistance has become a widespread phenomenon since its first discovery in the 1970s^1,3–5^. Indeed, the World Health Organization recently highlighted that infections caused by drug-resistant *A. baumannii* are one of the most dangerous threats to human society^6^.

Multidrug-resistant strains of *A. baumannii* harbour a genomic island containing up to 45 resistance genes, and sequence analysis suggests frequent exchange of genetic information between *A. baumannii* and other bacterial species^7^. Rapid acquisition of resistance has also been attributed to the high plasticity of protein expression in *A. baumannii*^8,9^. For example, loss of an outer membrane protein is associated with imipenem resistance^10,11^. The dynamic protein expression allows the species to exhibit various resistance mechanisms, including degradation of drugs, modification of protein targets, and production of efflux pumps^5,12,13^.

Multidrug efflux pumps play important roles in various bacterial interactions and maintenance of cell homeostasis^14^. These pumps have evolved long before the use of antibiotics, which is very recent in terms of bacterial evolution^15^. The pumps confer intrinsic, acquired, and induced resistance to antibiotics^16^. They also have wider roles in infection, such as increased pathogenicity through transport of virulence factors and aiding in biofilm formation^17^. Not surprisingly, overexpression of pumps has been documented in many clinical isolates^13,18^. In *A. baumannii*, basal-level expression of pumps is commonly observed and provides the bacteria with broad intrinsic resistance^12^. This low-level resistance allows for selection of mutations in the regulatory genes controlling expression, inducing higher-level, acquired resistance^19–21^.

Resistance-nodulation-division (RND) transporters are a major class of bacterial multidrug efflux pumps^14,22^. They have wide substrate scope and represent the highest clinical relevance in multidrug resistant bacteria^23–25^. At the molecular level, RND transporters have a tripartite structure, composed of an inner membrane protein, a membrane fusion protein, and an outer membrane protein. The three components interact with each other and form a functional transporter that extrudes a wide variety of toxic substrates from the cell^26,27^. In *A. baumannii*, RND-type efflux pumps have been shown to confer resistance to a wide range of antibiotics and detergents^5,19,28^. AdeABC was the first RND system characterised in *A. baumannii*^29^ and consists of the membrane fusion protein AdeA, inner membrane protein AdeB and outer membrane protein AdeC. Overexpression of *adeABC* was shown to increase the resistance to a wide range of substances^29–34^. This is also the case for two other RND pumps, AdeFGH and AdeIJK^33,34^. Interestingly, it was reported that a single membrane fusion protein, AdeT1, could also increase the antimicrobial susceptibility of *A. baumannii*^35^. In addition to the native effects in *A. baumannii*, heterologous expression of *adeABC*, *adeIJK* or *adeT1* in *Escherichia coli* have also been demonstrated to increase bacterial resistance to antibiotics^36^. Indeed, bacterial resistance proteins are often functional in heterologous hosts, and *E. coli* has been commonly used for functional characterisation of antibiotic resistance proteins from other bacteria^37–39^.

Intrigued by the function of AdeT1, here we show that overexpression of wildtype AdeT1, however, does not confer antimicrobial resistance in *E. coli*. We found that neither the plasmid nor the broth dilution method employed in the literature^35^ led to production of AdeT1 protein in *E. coli*. Using a new plasmid and the agar method, we were able to achieve consistent production of wildtype AdeT1 protein, which, in contrast to the literature^35^, did not confer antibiotic resistance to *E. coli*. Nevertheless, AdeT1 with propionylation at Lys280 confers a fourfold increase in MIC of erythromycin to *E. coli* BL21(DE3) cells.

## Results

### Constructing a plasmid for producing AdeT1 protein in *E. coli*

While working on site-specific protein post-translational modification in *A. baumannii*^40–42^, we found propionylation of AdeT1 at Lys280 (K280pr) in two clinical isolates of SK17-S and SK17-R^43^ (Supplementary Fig. S1). As *A. baumannii* AdeT1 was reported by Srinivasan et al. to enhance antimicrobial susceptibility^35^, we were interested in investigating whether this post-translational modification has functional significance. Particularly, post-translational modifications have been demonstrated to regulate different processes in bacteria^44–46^.

Since heterologous expression of AdeT1 was shown to confer antimicrobial resistance in *E. coli*^35^, we set out to establish this heterologous functional assay, which has two clear advantages. Unlike handling of *A. baumannii*, handling of a non-pathogenic *E. coli* strain poses minimum health and safety risks. In addition, genetic code expansion has been well established in *E. coli*, allowing site-specific incorporation of propionyl lysine for functional investigation of lysine propionylation^47,48^.

Specifically, Srinivasan et al. reported that *E. coli* KAM32 overexpressing *adeT1* increased bacterial resistance to various antibiotics^35^. This assertion was based on the minimum inhibitory concentration (MIC) values determined by broth dilution method. There, plasmid pAdeT1 was constructed from pUC18 for overexpressing *adeT1*, and MICs for erythromycin and chloramphenicol were reported to be 6- and 5-fold higher, respectively, in *E. coli* KAM32 carrying pAdeT1 than pUC18. *E. coli* KAM32 was chosen as it is hypersusceptible to antimicrobial agents due to the lack of major multidrug efflux pumps AcrB and YdhE^49^.

**Figure 1:**
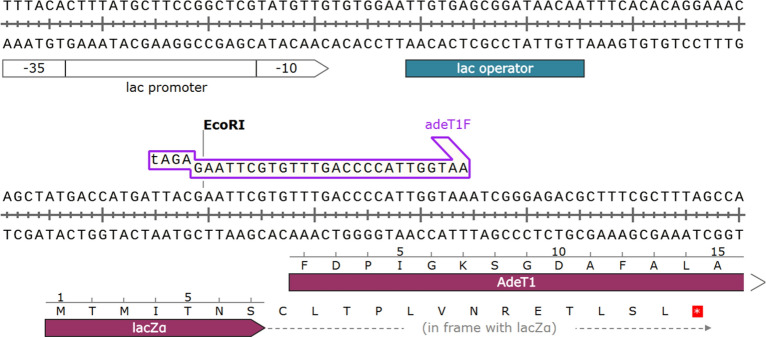
Extract of the plasmid map for the construct pAdeT1 employed by Srinivasan et al.^35^. In this construct, the *adeT1* gene is not in frame with a start codon or the open reading frame of lacZα. It is unlikely that this construct can lead to production of AdeT1 protein.

**Figure 2:**
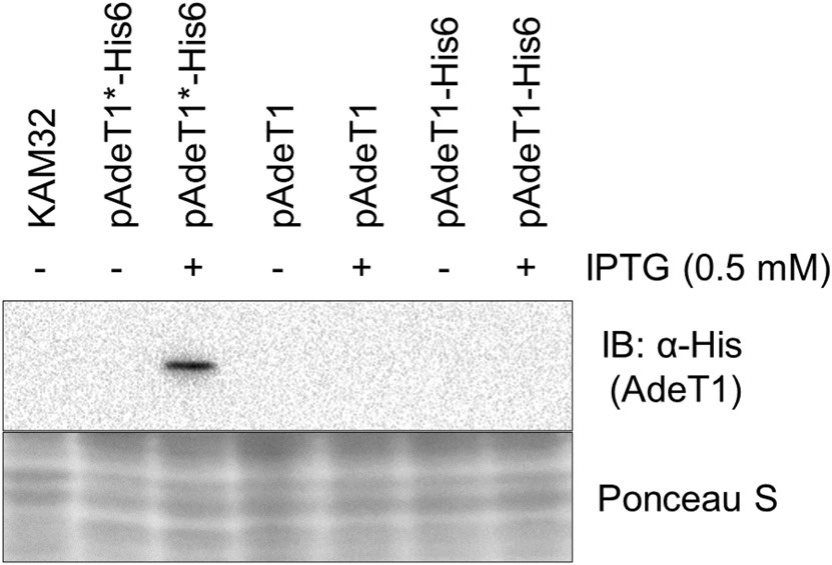
Immunoblotting analysis of AdeT1 expression in *E. coli* KAM32 from three plasmids. The pAdeT1 plasmid was constructed as described before^35^, while pAdeT1-His6 contains a 6 × Histidine tag on the C terminus of the protein encoding gene. The pAdeT1*-His6 plasmid is a similar construct, with the *adeT1* gene in frame with a start codon and with a 6 × Histidine tag on the C terminus. Cells were induced with 0.5 mM IPTG at OD_600_ ~ 0.6. After 2 h at 37 °C, samples were taken for analysis. A full-length version of the immunoblot is provided in Supplementary Fig. S3.

We followed the experimental procedure^35^ to construct pAdeT1 in house. As the *adeT1* gene is under the control of the lac promoter, we expected protein production to be induced by the addition of isopropyl β-d-1-thiogalactopyranoside (IPTG). However, we observed no AdeT1 protein in *E. coli* KAM32 carrying pAdeT1 when analysed by SDS-PAGE (Supplementary Fig. S2). Analysis of pAdeT1 indicated that the *adeT1* gene is not in frame with the open reading frame (Fig. 1). This could explain the lack of AdeT1 protein production, although we could not rule out the possibility of the protein produced at a low level, thus no prominent overexpression band in SDS-PAGE after Commassie Blue staining. To verify protein production via immunoblotting, which offers specificity and sensitivity over Commassie Blue staining, we constructed pAdeT1-His6, containing a His tag at the C-terminal of AdeT1 because there are no commercially available anti-AdeT1 antibodies. We also constructed pAdeT1*-His6, in which *adeT1* is in frame with the open reading frame. Not surprisingly, protein of the expected size was only detected with the plasmid pAdeT1*-His6 after IPTG induction but not under any other conditions (Fig. 2 and Supplementary Fig. S3). We therefore concluded that the literature reported plasmid, pAdeT1, is not functional for production of AdeT1 protein. Nevertheless, AdeT1 protein can be reproducibly produced in *E. coli* using plasmid pAdeT1*-His6.

**Figure 3:**
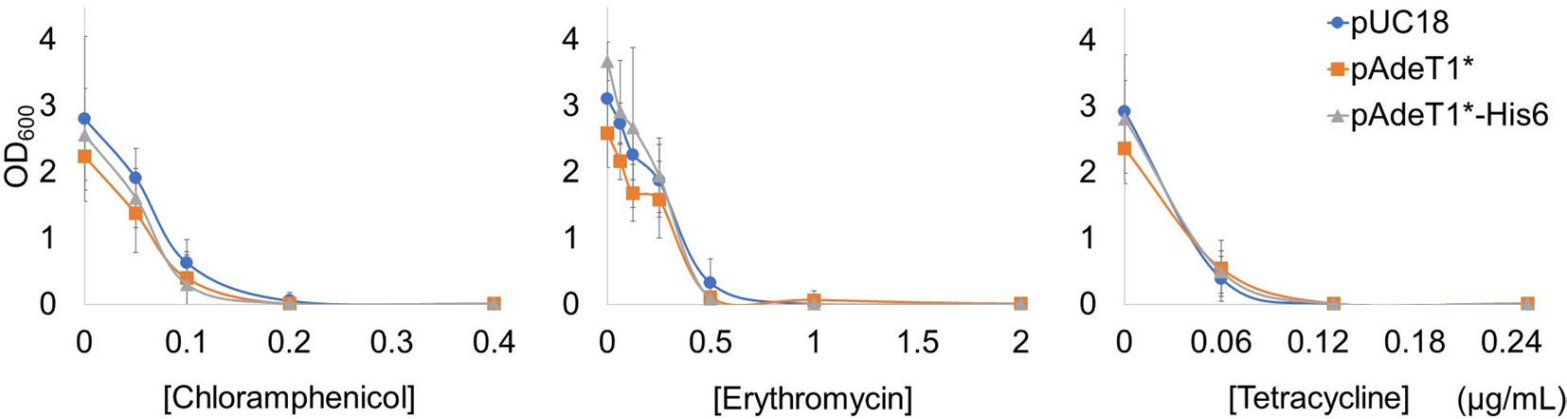
MIC testing of *E. coli* KAM32 cells carrying either pUC18, pAdeT1* or pAdeT1*-His6. Each data point represents an average of six biological replicates with the standard deviation. Optical density values were recorded on a Victor X (Perkin Elmer) microplate reader and converted to standard OD_600_ values using a calibration curve. Averages and standard deviations of the raw data are provided in Supplementary Tables S1–3, while the OD_600_ calibration curve is provided in Supplementary Fig. S4.

As AdeT1 protein was produced by pAdeT1*-His6, we also generated plasmid pAdeT1*, where the *adeT1* gene is in the correct reading frame, but without a 6 × histidine tag at the C-terminus. To confirm whether AdeT1 confers antimicrobial resistance in *E. coli*, we cultured *E. coli* KAM32 cells carrying either pUC18, pAdeT1* or pAdeT1*-His6 to OD_600_ 0.6, induced with IPTG, and waited 2 h after induction. The cells were then diluted into fresh media containing IPTG (to maintain protein production) and ampicillin (plasmid resistance marker) and underwent MIC testing in accordance with the CLSI microdilution standard^50^. Puzzlingly, *E. coli* KAM32 cells carrying either pUC18, pAdeT1* or pAdeT1*-His6 displayed no difference in MIC for chloramphenicol, erythromycin or tetracycline (Fig. 3 and Supplementary Tables S1–3 and Supplementary Fig. S4).

### Protein production through inducible promoters is not maintained after broth dilution

**Figure 4:**
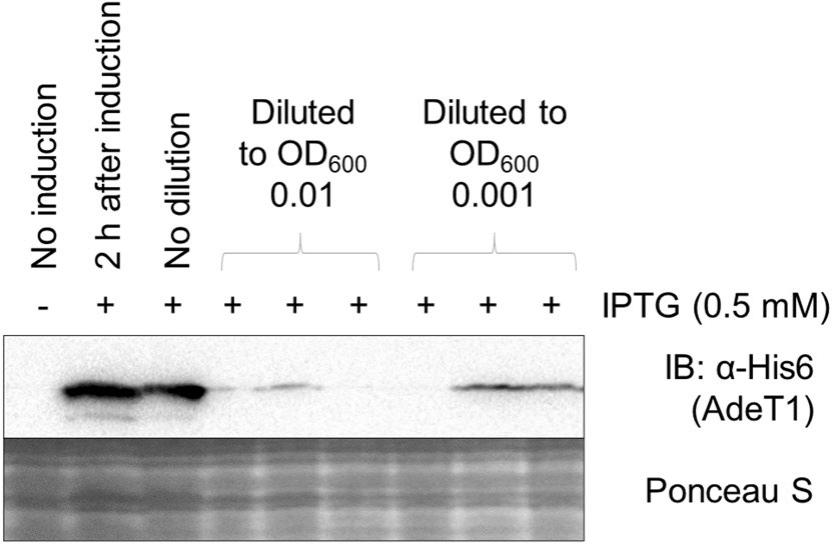
AdeT1 production was not always detectable after dilution of the induced culture to low optical density and subsequent overnight incubation in the presence of IPTG. *E. coli* KAM32 carrying pAdeT1*-His6 were induced with 0.5 mM IPTG at OD_600_ ~ 0.6. After 2 h, the induced culture was diluted to either OD_600_ 0.01 or 0.001 in fresh media containing 0.5 mM IPTG and 100 μg/mL ampicillin (for plasmid selection). Three technical replicates were prepared for each dilution. Full blots are shown in Supplementary Fig. S5.

As no difference in MIC was observed between control cells and those carrying pAdeT1* or pAdeT1*-His, we performed immunoblot analysis on cell samples from the end-point of MIC testing to confirm AdeT1 production. Cells carrying pAdeT1*-His6 were diluted to low optical density 2 h post-induction with IPTG and cultured overnight in media containing ampicillin and IPTG. Production of AdeT1 protein was then analysed via immunoblotting against the C-terminal His tag. Intriguingly, AdeT1 production was not always detectable after dilution to low optical density and subsequent overnight incubation (Fig. 4 and Supplementary Fig. S5). We were not able to identify a condition in which AdeT1 is always present in the diluted culture after overnight incubation (Supplementary Fig. S6).

We then constructed an alternative plasmid, pET28a AdeT1*-His6, in which *adeT1* is under the control of the commonly used T7 promoter. The T7 promoter induces high gene expression and we questioned whether this would allow more consistent protein production post induction. However, the lack of reproducible protein production after dilution was also observed with *E. coli* BL21(DE3) carrying pET28a AdeT1*-His6 (Supplementary Fig. S7).

To investigate if the lack of reproducible expression is unique to AdeT1 protein or a more generalised phenomena, we performed the same experiments with pET28a sfGFP where similar results were obtained (Supplementary Fig. S8). Thus, it appears that inducible lac and T7 promoters do not always work under dilution conditions. Attempts to determine MIC without prior dilution of the culture were not successful. Although protein was consistently detected after overnight incubation with antimicrobials, it was difficult to determine whether growth was inhibited or not (Supplementary Fig. S9).

### Constitutively active promoters are not compatible for production of AdeT1

**Figure 5:**
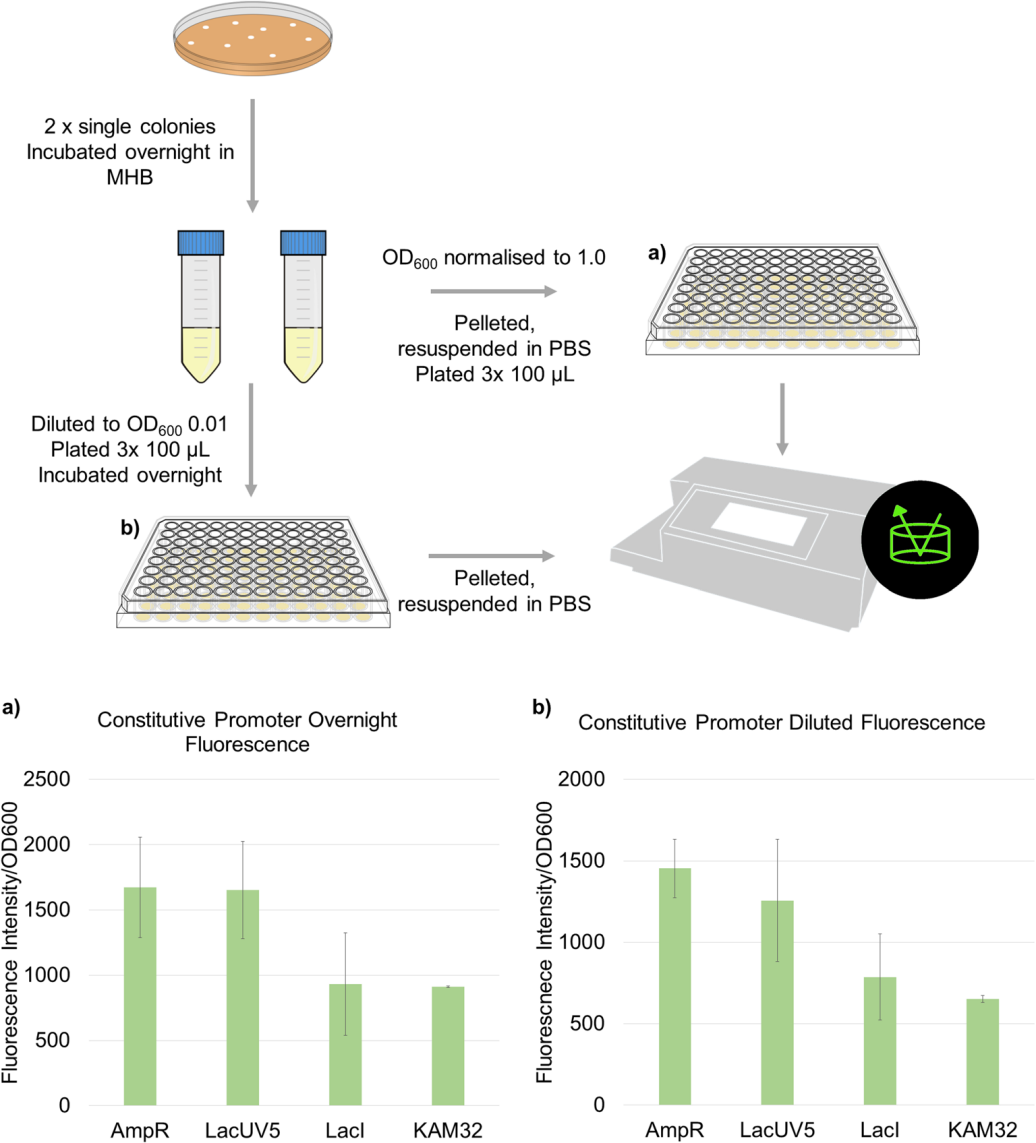
Fluorescence intensity of *E. coli* KAM32 cells containing *sfGFP* under the control of three constitutive promoters: AmpR, LacUV5 and LacI. Fluorescence was measured after incubation at 37 °C for 18 h (**a**), or after dilution of that culture to OD_600_ 0.01 and further incubation for another 18 h (**b**). Averages and standard deviations of two transformations, each with three technical replicates, are shown.

**Figure 6:**
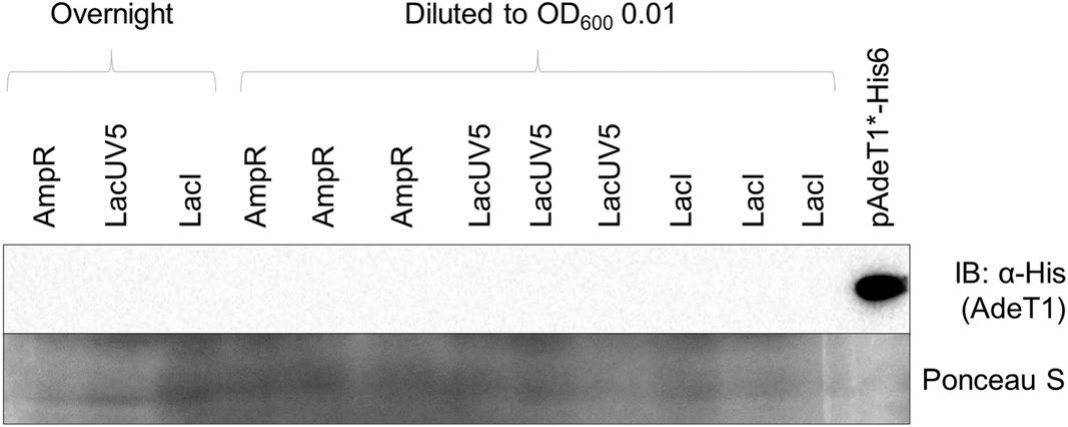
Immunoblotting analysis of AdeT1 production under the control of constitutively active promoters. A single colony was cultured overnight in MHB, then diluted to OD_600_ 0.01 with three replicates and cultured overnight Full blots are shown in Supplementary Fig. S10.

We envisaged that a constitutive promoter could address this problem. Thus, we constructed plasmids pAmpR-sfGFP, pLacUV5-sfGFP and pLacI-sfGFP, in which expression of *sfGFP* is controlled by the constitutively active ampicillin resistance gene (AmpR) promoter, the LacUV5 promoter or the lac repressor gene (LacI) promoter, respectively. The LacUV5 promoter was made constitutively active through removal of the regulatory lac operator. *E. coli* KAM32 carrying a plasmid, in which *sfGFP* is under the control of AmpR or LacUV5 promoter, showed significantly higher green fluorescence (*p* < 0.05) than the wild-type cells (Fig. 5). In addition, the green fluorescence persisted in the diluted culture after overnight incubation. Puzzlingly, when *sfGFP* was directly replaced with adeT1*-His6, no His-tagged protein was detectable by immunoblotting (Fig. 6 and Supplementary Fig. S10).

### AdeT1 forms an active efflux pump in *E. coli*

**Figure 7:**
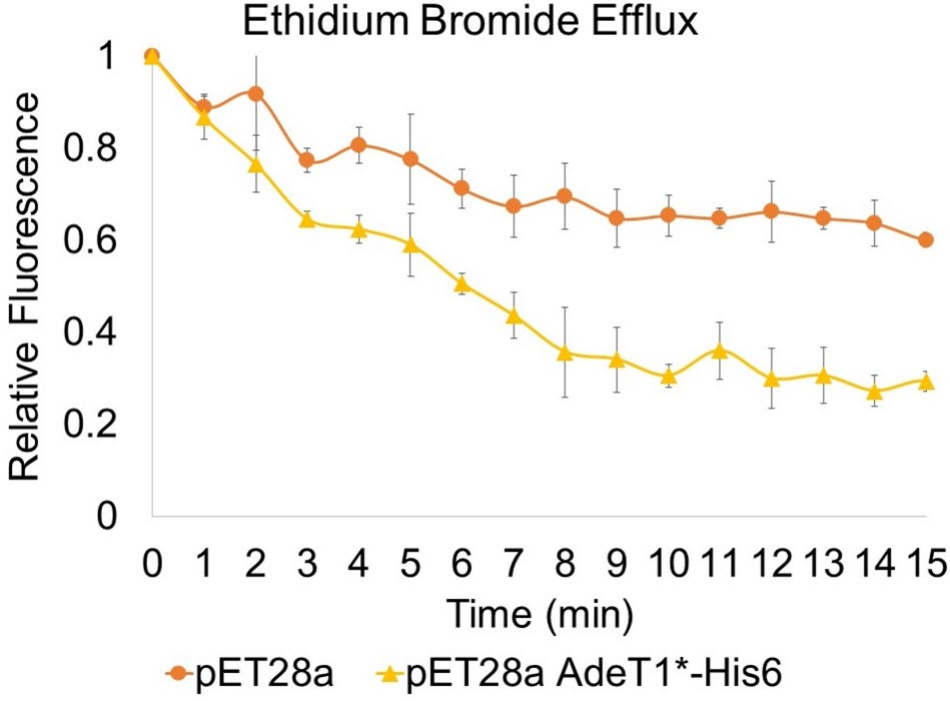
Efflux of ethidium bromide from *E. coli* BL21(DE3) cells expressing pET28a or pET28a AdeT1*-His6. Each datapoint represents an average of three technical replicates, standard deviation is annotated for each average. The data shown is a representative example among four biological replicates. Results of the other biological replicates are shown in Supplementary Fig. S11.

To confirm whether AdeT1 protein forms an active efflux pump in *E. coli*, we performed a dye-based efflux assay with ethidium bromide^51^. As shown in Fig. 7 and Supplementary Fig. S11, a significantly greater reduction in fluorescence can be observed in *E. coli* BL21(DE3) cells containing pET28a AdeT1*-His6 in comparison to control cells. For *E. coli* KAM32 cells, a similar trend was observed, however, the difference between control and AdeT1-producing cells was less pronounced and not significantly different, likely due to the relatively smaller amount of AdeT1 protein produced from the pAdeT1*-His6 vector compared to pET28a AdeT1*-His6. Nevertheless, the results of the efflux assays suggest that the AdeT1 protein forms an active efflux pump in *E. coli* cells and is involved in export of ethidium bromide.

### Production of wildtype AdeT1 does not increase the antimicrobial susceptibility of *E. coli* KAM32 or BL21(DE3)

**Figure 8:**
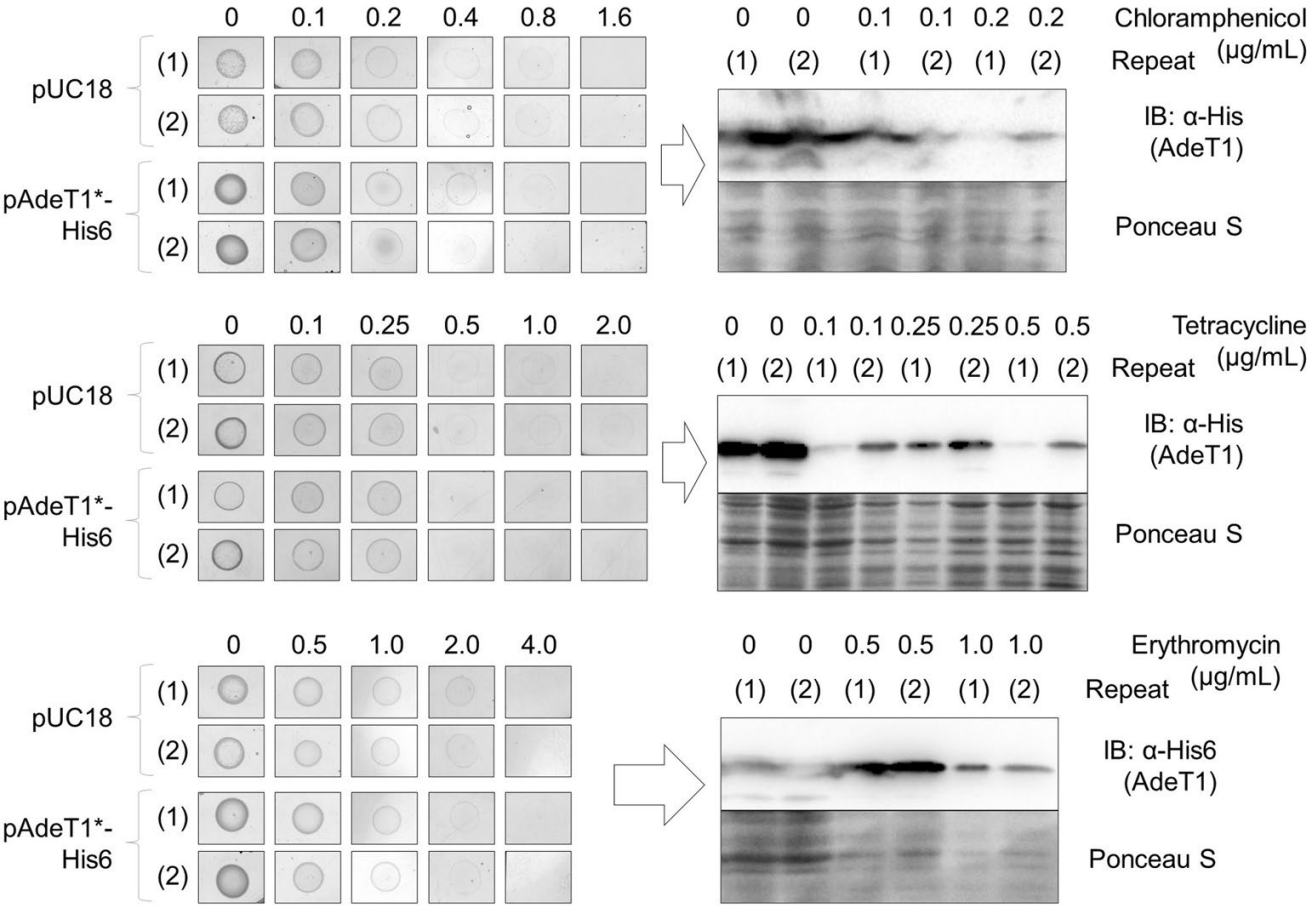
Antibiotic susceptibility of *E. coli* KAM32 carrying either pUC18 or pAdeT1*-His6. Data of two biological replicates (i.e. independent transformations), (1) and (2), are shown. *E. coli* KAM32 carrying either pUC18 or pAdeT1*-His6 were cultured to OD_600_ 0.6 before induction with 0.5 mM IPTG. After 2 h, a defined amount of cells were dropped on agar plates containing 0.5 mM IPTG, 100 µg/mL ampicillin and varying concentrations of chloramphenicol (top), tetracycline (middle) or erythromycin (bottom). Plates were incubated at 37 °C for 18 h before colonies of *E. coli* KAM32 carrying pAdeT1*-His6 were analysed by immunoblotting to confirm protein expression. Full-length blots are shown in Supplementary Figs. S12 and S13.

Due to the difficulty of maintaining protein production in diluted liquid cultures, we attempted to determine MIC using agar plates. As standard agar dilution methods^52^ require dilution to low optical density, we modified the procedure and used a high density sample of bacterial cells. After IPTG induction for 2 h, a defined amount of *E. coli* KAM32 cells carrying pAdeT1*-His6 were concentrated to OD_600_ 4.0 and dropped onto agar plates containing IPTG, ampicillin, and varying concentrations of the test antibiotic (i.e. chloramphenicol, tetracycline or erythromycin). With this method, all colonies on the agar plates showed detectable and reproducible quantities of AdeT1 by immunoblotting. However, no difference in MIC was observed between *E. coli* KAM32 carrying pAdeT1*-His6 and *E. coli* KAM32 carrying pUC18 for either chloramphenicol, tetracycline or erythromycin (Fig. 8 and Supplementary Figs. S12 and S13). Furthermore, *E. coli* BL21(DE3) carrying either pET28a AdeT1*-His6 or pET28a also demonstrated no difference in antimicrobial susceptibility (Supplementary Figs. S14 and S15).

### Production of propionylated AdeT1 decreases the antimicrobial susceptibility of *E. coli* BL21(DE3) to erythromycin

As AdeT1 can be propionylated at Lys280 in vivo (Supplementary Fig. S1), we investigated whether this post-translational modification had any effect on antimicrobial resistance in the *E. coli* model. AdeT1 with propionylated Lys280, AdeT1(K280pr), can be generated by genetic code expansion^47,48^. In genetic code expansion, an orthogonal aminoacyl-tRNA synthetase/tRNA pair is introduced into the cell^53^. The orthogonal synthetase recognises a specific unnatural amino acid, propionyl lysine (PrK) in this instance, and loads it onto the orthogonal tRNA. The orthogonal tRNA is engineered to decode the amber stop codon (UAG). Thus, by mutating the codon of the corresponding amino acid residue to the amber codon, site-specific incorporation of PrK can be achieved^47,48^.

Thus, we mutated the Lys280 codon to TAG in the *adeT1* gene of both pAdeT1*-His6 and pET28a AdeT1*-His6 to generate pAdeT1*(K280TAG)-His6 and pET28a AdeT1*(K280TAG)-His6, respectively. Each construct was provided to the cells alongside a plasmid containing the corresponding orthogonal aminoacyl-tRNA synthetase/tRNA_CUA_ pair, and PrK was supplemented into the growth media at the point of IPTG induction. Initial expression tests indicated that production of full-length AdeT1 protein containing PrK was more effective in *E. coli* BL21(DE3) cells with pET28a AdeT1*(K280TAG)-His6 than KAM32 cells with pAdeT1*(K280TAG)-His6 (Supplementary Fig. S16).

While a chemiluminescent signal was detected in samples which were not provided with PrK (Supplementary Fig. S16), such signals were also detected when expressing sfGFP(150TAG) in the absence of propionyl lysine (Supplementary Fig. S17). Thus, the presence of full-length protein in the absence of propionyl lysine is likely the result of low-level incorporation of a canonical amino acid. Nevertheless, mass spectrometry confirmed the presence of PrK in AdeT1 in samples supplemented with the unnatural amino acid, and no wildtype or other species were observed (Supplementary Fig. S18).

**Figure 9:**
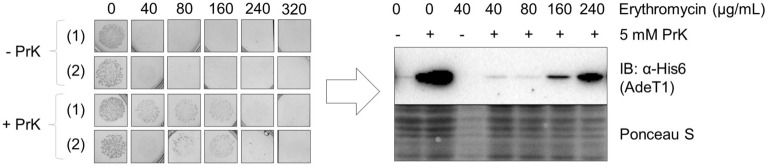
Antibiotic susceptibility of *E. coli* BL21(DE3) carrying pET28a AdeT1*(K280TAG)-His6 expressed either with or without propionyl lysine (PrK). Data of two biological replicates/independent transformations, (1) and (2), are shown. *E. coli* BL21(DE3) carrying pET28a AdeT1*(K280TAG)-His6 were cultured to OD_600_ 0.6 before induction with 0.5 mM IPTG and supplementation with 5 mM PrK (as appropriate). After 2 h, a defined amount of cells were dropped on agar plates containing 0.5 mM IPTG, 5 mM PrK, 100 µg/mL spectinomycin, 50 µg/mL kanamycin and varying concentrations of erythromycin. Plates were incubated at 37 °C for 18 h before colonies were analysed by immunoblotting to confirm protein expression. Full length blots are shown in Supplementary Fig. S19.

Agar-based MIC tests were performed as described earlier with *E. coli* BL21(DE3) cells and the pET28a AdeT1*(K280TAG)-His6 construct. Intriguingly, the MIC of erythromycin required was fourfold higher when PrK was provided to the cells than the control (320 µg/mL and 80 µg/mL, respectively) (Fig. 9 and Supplementary Fig. S19). No difference in MIC was observed for tetracycline nor chloramphenicol (Supplementary Fig. S20). Two further antibiotics, ertapenem and ampicillin, were also tested and no difference in MIC was observed (Supplementary Fig. S20).

## Discussion

An RND transporter has three components: an inner membrane protein, a membrane fusion protein, and an outer membrane protein^54^. Membrane fusion proteins, such as AdeT1, alone are not functional for drug efflux. Nevertheless, components of an RND transporter can be functional in a heterologous host. For example, two components of *Haemophilus influenzae* multidrug efflux pump AcrAB were found to function with the *E. coli* outer membrane protein TolC^55^. Similarly, *Pseudomonas aeruginosa* multidrug efflux pump MexXY can also assemble with *E. coli* TolC for drug efflux^56^. In addition, *P. aeruginosa* MexB is an inner membrane protein that can complex with *E. coli* AcrA and TolC to form a tripartite pump^57^. Thus, it would not be a surprise if AdeT1 can form a functional pump with endogenous *E. coli* RND pump components as indicated in the literature^35^. However, our results clearly showed that production of AdeT1 in *E. coli* did not enhance the bacterial resistance to either chloramphenicol or erythromycin (Fig. 8).

Constitutively active promoters have been successfully, albeit infrequently, employed in *E. coli* for producing recombinant proteins^58–63^. We demonstrated that two constitutive promoters allowed constant production of sfGFP at low levels (i.e., no obvious green fluorescence in cultures by visual observation, in contrast to sfGFP under T7 promoter). However, we could not successfully produce AdeT1 using the same promoters, and the reason remains elusive.

The biggest obstacle we faced was maintenance of protein production after diluting post-induction cells to low optical densities. It was reported that dilution of *E. coli* BL21(DE3) may lead to chromosomal mutations, resulting in impaired production of functional T7 RNA polymerase^64^. This could explain why our protein of interest was not reproducibly detected in diluted cultures.

Plasmid pAdeT1 employed by Srinivasan et al.^35^ for MIC determination also had a sequence frame-shift problem which was addressed in this report. Use of the agar method for MIC determination resulted in consistent production of wildtype AdeT1 protein, which forms a functioning efflux pump capable of ethidium bromide efflux, but did not confer resistance in *E. coli* KAM32 or BL21(DE3) to either erythromycin, chloramphenicol or tetracycline. It is noteworthy that the commonly used broth dilution method in MIC determination may not frequently produce target protein with an inducer, which may cause unreliable MIC results. Therefore, we strongly recommend the confirmation of target protein expression when investigating relationships between target proteins and drug resistance.

Finally, AdeT1 protein is propionylated in *A. baumannii *in vivo. When propionylated AdeT1 protein is produced by *E. coli* BL21(DE3) cells, a fourfold increase in the MIC of erythromycin is observed. Such an increase in MIC was not observed for four other antibiotics tested. We therefore preliminarily report that propionylation may regulate the function of AdeT1 protein. However, it is important to note that only AdeT1 protein was heterologously produced in *E. coli*. Presumably, in *A. baumannii* the AdeT1 protein forms an efflux pump complex with other RND-type proteins. When expressed in the heterologous *E. coli* host, AdeT1 may assemble with endogenous *E. coli* efflux pump components to form a pump complex which is not found in vivo^55–57^.

Therefore, the mechanism of erythromycin resistance in this instance may be a consequence of a pump complex which does not form in nature, and propionylation of AdeT1 protein may not directly relate to erythromycin resistance in *A. baumannii*. Nevertheless, under these conditions, propionylation was able to modulate AdeT1 function. Our results provide evidence in agreement with the growing body of literature reporting that in vivo post-translational modifications regulate protein function.

## Methods

### Plasmid construction

Plasmid pBAD sfGFP was a kind gift from Dr D. Dafydd Jones. The pET28a control plasmid for pET28a AdeT1*-His6 in our studies is plasmid pET28a sfGFP(150TAG) (Addgene plasmid #133455). After IPTG induction, a 16.8 kDa protein is produced. Plasmids used in this study are listed in Table 1. Tables 2 and 3 list primers for PCR and sequencing, respectively.

**Table 1:** Plasmids used in this study.

Plasmid	Vector	Insert	Antibiotic selection	Cloning sites	Notes
pAdeT1	pUC18	*adeT1*	Ampicillin	EcoRI, BamHI	*adeT* gene is not in frame with start codon
pAdeT1-His6	pUC18	*adeT1*	Ampicillin		
pAdeT1*	pUC18	*adeT1*	Ampicillin		
pAdeT1*-His6	pUC18	*adeT1*	Ampicillin	BamHI, EcoRI	
pET28a	pET28a	*sfGFP *(150TAG)	Kanamycin		Induction of this gene results in truncated sfGFP (16.8 kDa)
pET28a AdeT1*-His6	pET28a	*adeT1*	Kanamycin	BamHI, NcoI	
pET28a sfGFP-His6	pET28a	*sfGFP*	Kanamycin	NcoI, XhoI	
pAmpR-sfGFP-His6	pET21a	*sfGFP*	Ampicillin		
pLacUV5-sfGFP-His6	pET21a	*sfGFP*	Ampicillin		LacUV5 promoter is constitutively active through removal of lac operator
pLacI-sfGFP-His6	pET21a	*sfGFP*	Ampicillin		
pAmpR-AdeT1-His6	pET21a	*adeT1*	Ampicillin	NcoI, XhoI	
pLacUV5-AdeT1-His6	pET21a	*adeT1*	Ampicillin	NcoI, XhoI	LacUV5 promoter is constitutively active through removal of lac operator
pLacI-AdeT1-His6	pET21a	*adeT1*	Ampicillin		
pAdeT1*(K280TAG)-His6	pUC18	*adeT1 *(K280TAG)	Ampicillin		
pET28a AdeT1*(K280TAG)-His6	pET28a	*adeT1 *(K280TAG)	Kanamycin		
pAcKST		*Mb*AcKRS, Pyl tRNA	Spectinomycin		MbAcKRS is a variant of MbPylRS with OOXX mutations

**Table 2:** Primers for PCR.

Primer	Sequence (5′–3′)
VBF004	GTTATTGTCTCATGAGCGGATACATATTTGAATGTATTTAGAAAAATAAACAAATAGGGGTTCCGCGGATCCAAGGAGGAACTATATCCGGATTGGCGAATGG
VBR004	TAAAGCTCGAGATCTGCAGCTGGCGCAACGCAATTAATGTAAGTTAGC
VBF005	CCAGCTGCAGATCTCGAGCTTTAATG
VBR005	GTATCCGCTCATGAGACAATAACCCTGATAAATGCTTCAATAATATTGAAAAAGGAAGAGTCCATGGTTAGCAAAGGTGAAGAACTG
VBF007	CAACATACGAGCCGGAAGCATAAAGTGTAAAGAATTCAAAGGAGGAACTATATCCGGATTGG
VBR007	TTTACACTTTATGCTTCCGGCTCGTATGTTGCCATGGTTAGCAAAGGTGAAGAACTG
VBF015	ACATTATACGAGCCGGAAGCATAAAGTGTAAAGCCTGGGGTGCCTAATGAGTGAGAATTCAAAGGAGGAACTATATCCGGATTGG
VBR015	GCTTCCGGCTCGTATAATGTGTGGAAAAGCTTGGATCCCATGGTTTCACACAGGAAACAGCTATGGTTAGCAAAGGTGAAGAACTG
VBF016	GACACCATCGAATGGCGCAAAACCTTTCGCGGTATGGCATGATAGCGCCCGGAAGAGAGTCAATTCAGGGTGGTGAATATGGTTAGCAAAGGTGAAGAACTG
VBR016	TTGCGCCATTCGATGGTGTCGAATTCAAAGGAGGAACTATATCCGGATTGG
VBR019	CGTTGCGCCAGCTGCAGATCTCGAGCTTCAGTGGTGGTGATGATGATGTTCATCGTTCAGG
VBF020	TGCTTCAATAATATTGAAAAAGGAAGAGTCCATGTTTGATCCGATTGGTAAAAGCGGTG
VBR020	GCGCCAGCTGCAGATCTCGAGCTTTAGTGGTGGTGATGATGATGTTCATCGTTCAGG
VBF021	GTGTGGAAAAGCTTGGATCCCATGGTTTCACACAGGAAACAGCTATGTTTGATCCGATTGGTAAAAGCGGTGATGC
VBF022	GACACCATCGAATGGCGCAAAACCTTTCGCGGTATGGCATGATAGCGCCCGGAAGAGAGTCAATTCAGGGTGGTGAATATGTTTGATCCGATTGGTAAAAGCGGTGATGC
VBR025	GGGATCATAAATACCTGCTTTGGTC
VBF027	AAGCAGGTATTTATGATCCCTAGATGATGAACTTCCTGAAGAAAGTGC
VBR033	TGCAGGTCGACTCTAGAGTCATTCATCGTTCAGGGCACATTC
VBF035	TGACTCTAGAGTCGACCTGCAG
SPF001	TTCATCATCTAGGGATCATAAATACCTGCTTTGGTCAG
SPR001	TATGATCCCTAGATGATGAACTTCCTGAAGAAAGTGC

**Table 3:** Primers for sequencing.

Primer	Sequence (5′–3′)
YTS14	CCGATTCTGGTGGAACTG
YTS15	TAGGTCAGGGTGGTCAC
YTS30	ATGGTGTCCGGGATCTC
YTS47	GCCTTTTGCTCACATGTTC
YTS52	TTAATGCGCCGCTACAG
YTS53	AAATACCGCACAGATGC
VBS014	CATTGGTCCGGCATACC
VBS017	CAATCGGTGCCGGAAC

For cloning of pAdeT1, AdeT1 in a pUC18 vector was constructed as described by Bharathi Srinivasan et al.^35^ The sequence was confirmed using primers YTS47 and YTS53.

For cloning of pAdeT1-His6, site-directed mutagenesis was performed to insert a 6 × Histidine tag at the C-terminus of the *AdeT1* gene in pAdeT1 to construct pAdeT1-His6. The resulting sequence was confirmed using primers YTS47 and YTS53.

For cloning of pAdeT1*-His6, the *adeT1* gene was amplified by PCR. The resulting 973 bp fragment was cloned into a pUC18 vector linearised with restriction enzymes EcoRI and BamHI using NEBuilder (New England BioLabs, #E2621S) to afford pAdeT1*-His6. The plasmid sequence was confirmed using primers YTS47 and YTS53.

For cloning of pAdeT1*, a 3597 bp fragment was amplified from plasmid pAdeT1*-His6 using primers VBF035 and VBR033. The resulting sequence was confirmed using primers YTS47 and YTS53.

For cloning of pET28a sfGFP-His6, both pET28a and pBAD sfGFP were digested with NcoI and XhoI to afford the vector and insert, respectively. The two fragments were assembled by T4 ligation. The resulting plasmid was sequenced with T7 promoter and T7 terminator primers.

For cloning of pET28a AdeT1*-His6, a 1000 bp fragment was PCR amplified and inserted into pET28a digested with BamHI and NcoI using NEBuilder (New England BioLabs, #E2621S). The resulting plasmid was sequenced with primers YTS30 and YTS52.

For cloning of pAmpR-sfGFP-His6 that constitutively expresses sfGFP by the AmpR (ampicillin resistance gene) promoter, a 3762 bp vector fragment was PCR amplified from pET21a using primers VBF004 and VBR004, and an 827 bp fragment containing sfGFP was PCR amplified from pBAD sfGFP using primers VBF005 and VBR005. The two fragments were assembled using NEBuilder (New England BioLabs, #E2621S) to afford pAmpR sfGFP, which was confirmed by sequencing with primers YTS14 and YTS15.

For cloning of pLacUV5-sfGFP-His6, a 3728 bp vector fragment was amplified from pET21a using primers VBF007 and VBR004. A 797 bp fragment was amplified from pBAD sfGFP using primers VBF005 and VBR007. The two fragments were assembled using NEBuilder (New England BioLabs, #E2621S). The resulting plasmid was subjected to site-directed mutagenesis with primers VBF015 and VBR015. The final plasmid was sequenced using primers YTS14 and YTS15.

For cloning of pLacI-sfGFP-His6, a 4536 bp fragment was amplified using primers VBF016 and VBR016 and directly transformed into *E. coli* Stbl3 where homologous recombination afforded plasmid pLacI-sfGFP-His6. The plasmid sequence was confirmed using primers YTS14 and YTS15.

For cloning of pAmpR-AdeT1-His6, the *adeT1* gene was cloned downstream of the constitutively active ampicillin resistance promoter by replacing the *sfGFP* gene in pAmpR-sfGFP-His6 with *AdeT1*. The *AdeT1* gene was amplified via PCR using primers VBF020 and VBR019. The resulting 991 bp fragment was inserted into pAmpR-sfGFP-His6 digested with restriction enzymes XhoI and NcoI and assembled using NEBuilder (New England BioLabs, #E2621S). The resulting plasmid, pAmpR-AdeT1-His6, was sequenced using primers VBS014 and VBS017.

For cloning of pLacUV5-AdeT1-His6, the *AdeT1* gene was amplified using primers VBF021 and VBR020 and the resulting 1000 bp fragment was inserted into pLacUV5-sfGFP-His6 plasmid digested with restriction enzymes NcoI and XhoI. The two fragments were assembled using NEBuilder (New England BioLabs, #E2621S) and the resulting plasmid sequenced using primers VBS014 and VBS017.

For cloning of pLacI-AdeT1-His6, the *AdeT1* gene was amplified using primers VBF022 and VBR020, resulting in a 1034 bp fragment and the pET21a vector was amplified using primers VBR016 and VBR004 to result in a 3717 bp plasmid. The two fragments were assembled using NEBuilder (New England BioLabs, #E2621S) and the resulting plasmid was sequenced using primers VBS014 and VBS017.

For cloning of pAdeT1*(K280TAG)-His6, site-directed mutagenesis was used to mutate the lysine codon at position 280 to the amber stop codon (TAG) using forward primer VBF027 and reverse primer VBR025. The resulting plasmid was sequenced with primers VBS014 and VBS017.

For cloning of pET28a AdeT1(K280TAG)-His6, site directed mutagenesis was used to construct plasmid pET28a AdeT1(K280TAG)-His6 using forward primer SPF001 and reverse primer SPR001 with pET28a AdeT1-His6 as a template. The resulting plasmid was sequenced with primers YTS30 and YTS52.

### Identification of AdeT1 propionylation

Data^41^ obtained from the acetylome studies of *A. baumannii* SK17-S and SK17-R were analysed for lysine propionylation using the procedure described before^42^.

### Expression trials

The bacterial strain *E. coli* TG1 KAM32 (*ΔacrB, ΔydhE*) was used for this study and provided as a kind gift from Professor Teruo Kuroda, Hiroshima University. *E. coli* BL21(DE3) cells were also used when T7 expression was required.

### Efflux assays

Ethidium bromide efflux assays were performed as described by Viveiros et al.^51^ with 0.5 mM IPTG added to cultures at OD_600_ 0.6 before centrifugation, and included in the PBS used during ethidium bromide accumulation. For the efflux pump inhibitor, 100 µg/mL 1-(1-naphthylmethyl)-piperazine was used. Each experiment contains three technical replica for each condition, and four biological repeats of each experiment were conducted. Fluorescence measurements were obtained with a FLUOStar (Optima) plate reader (ex 500, em 590, gain 4095).

### Inducible expression

For all expressions, a starter culture was prepared by inoculating a single colony into 5 mL Mueller Hinton Broth (MHB) (Sigma-Aldrich #70192) + 100 µg/mL ampicillin or 50 µg/mL kanamycin, as appropriate, and at 37 °C, 180 rpm for 18 h. The starter culture was then diluted in fresh MHB + 100 µg/mL ampicillin/50 µg/mL kanamycin to an OD_600_ of 0.05 and incubated at 37 °C, 180 rpm. At OD_600_ 0.6, cultures were induced with 0.5 mM IPTG and incubated for 1–2 h at 37 °C, 180 rpm before use in MIC testing.

### MIC tests

Microdilution studies were performed in accordance with CLSI microdilution protocols^50^. Specifically, cells were diluted to OD_600_ 0.02 in fresh MHB containing 100 µg/mL ampicillin and 0.5 mM IPTG. This inoculum (50 ﻿μL) was then added to a 96 well microplate, with each well containing 50 µL of MHB containing 100 µg/mL ampicillin, 0.5 mM IPTG and varying concentrations of the test antibiotic. Plates were incubated at 37 °C for 16–20 h before MIC was determined. MIC is defined as the minimum concentration of antibiotic required to visually inhibit bacterial growth as observed by the unaided eye. Visual observations were further supported by measurement of the OD_600_ of each well on a Victor X (Perkin Elmer) plate reader.

When MIC was tested without prior dilution of the culture, the culture was divided into 10 mL fractions 2 h post-induction, and chloramphenicol added directly. The culture was then incubated for 18 h at 37 °C, 180 rpm before OD_600_ was measured.

For agar MIC methods, the OD_600_ of 2 h post-induction cultures were ~ 1–1.2. At this point, the cultures were normalised OD_600_ 4.0. This was achieved by pelleting the cells and removing some of the supernatant so that when resuspended, the cells would be the required OD_600_. Then, 20 µL of these adjusted cultures were dropped onto LB agar plates containing 0.5 mM IPTG, 100 µg/mL ampicillin or 50 µg/mL kanamycin as appropriate, and various concentrations of test antibiotic, and incubated at 37 °C for 18 h.

### Propionyl lysine incorporation

Appropriate plasmid containing *adeT1* gene with a TAG codon mutation was co-transformed with a plasmid containing acetyl lysine synthetase and 1 copy of a corresponding tRNA (pAcKST). MIC testing was performed as before, with 5 mM PrK added to the culture at the point of induction with IPTG, and included in the antibiotic-testing agar plates.

### Constitutive expression

A single colony was used to inoculate 5 mL MHB containing 100 µg/mL ampicillin and incubated for 18 h at 37 °C, 180 rpm. The culture was then diluted to OD_600_ 0.01 into 5 mL fresh MHB containing 100 µg/mL ampicillin and 100 µL of this dilution was plated in triplicate into a 96-well plate and incubated for 18 h at 37 °C, 180 rpm.

### Immunoblotting analysis

Samples from liquid cultures were taken by normalising the OD_600_ to 1.5 and taking 1 mL. Cells were then pelleted and resuspended in 50 µL of SDS loading dye (50 mM Tris–Cl (pH 6.8); 2% (w/v) sodium dodecyl sulfate; 0.1% (w/v) bromophenol blue; 10% (v/v) glycerol; 100 mM dithiothreitol). For low volume liquid cultures where normalising the OD_600_ was not possible, the whole culture was pelleted and resuspended in 10 µL loading dye. For solid cultures from agar plates, the whole colony was resuspended in phosphate buffered saline (PBS, 50 mM NaPi, 150 mM NaCl pH 7.4), pelleted and resuspended in 10–50 µL loading dye depending on cell density of colony.

Samples were heated (95 °C, 5 min) and 10 µL of all samples were loaded onto 12% SDS-PAGE and electrophoresed with 155 V, 55 mA, for 1 h. The gel was transferred to a 0.2 µM nitrocellulose membrane (#1704158) using a Trans-Blot Turbo Transfer System (BioRad #1704150) with mixed molecular weight setting applied. Membrane was then stained with Ponceau S (0.1% Ponceau S in 5% acetic acid), then blocked in 5% (w/v) milk (Sigma-Aldrich #70166) PBST (0.05% (v/v) Tween 20 in PBS) for 1–2 h at 18 °C with gentle agitation. Membrane was then transferred to 5% milk PBST containing primary mouse 6x-His tag monoclonal antibody (ThermoFisher #MA121315, 1:1000 (v/v) dilution) and incubated overnight at 4 °C. Membrane was then washed for 5 min in PBST 3 times, before incubation with 5% milk PBST containing goat anti-mouse IgG (H + L) secondary antibody (ThermoFisher #32430, 1:1000 (v/v) dilution) for 1–2 h at 18 °C with gentle agitation. Membrane was then washed for 5 min in PBST 3 times. Signal was developed using Clarity Max (Bio-Rad #1705062) and imaged using a ChemiDoc XRS + system (Bio-Rad #1708265).

### Fluorescence intensity measurements

Overnight samples were taken by normalising the OD_600_ to 1.5 and taking 1 mL of culture, which was pelleted and resuspended in 1 mL PBS. For each sample, 100 µL was loaded in triplicate onto a 96 well plate (Fisher Scientific #167008). Samples from cultures grown in a 96-well plate were taken by centrifuging cultures in plate, discarding supernatant and resuspending in 100 µL PBS. OD_600_ was measured on a Victor X (Perkin Elmer) plate reader using the OD_600_ protocol (CW-lamp OG590, filter B7) with 5 s shaking before measurement. Fluorescence intensity was measured on FLUOstar (Optima) plate reader (ex 485, em 520, gain 804).

### Equipment and settings

All images were taken with a ChemiDoc XRS + system (Bio-Rad #1708265) and processed using Image Lab software (Biorad). For MIC testing, pictures of colonies were taken using the white light conversion screen and all images were adjusted identically to enhance the contrast and visibility of colonies by using the ‘Image Transform’ tool and setting ‘High’ to 41,215 and ‘Low’ to 8192. For immunoblots, images of Ponceau S stains may have contrast adjusted to increase band visibility. Images of chemiluminescent signal have not had any adjustment made although may be cropped in the main manuscript. Original, uncropped versions of all blots can be found in the Supplementary Information.

## Supplementary information

Supplementary Information.

## Data Availability

All data generated or analysed during this study are included in this published article and its Supplementary Information file (Supplementary Figs. S1–S20). Information on the original data underpinning the results presented here, including how to access them, can be found in the Cardiff University data catalogue at 10.17035/d.2020.0120368784.
