# Supplementary material for: Effect of membrane fusion protein AdeT1 on the antimicrobial resistance of Escherichia coli
Source: Sci Rep. 2020 Nov 24;10:20464. doi: 10.1038/s41598-020-77339-w (PMC7687900; doi:10.1038/s41598-020-77339-w)
Supplement: Supplementary file 1 — Supplementary Information. [file 41598_2020_77339_MOESM1_ESM.docx]

# Effect of membrane fusion protein AdeT1 on the antimicrobial resistance of *Escherichia coli*

Victoria L. Barlow^1^, Shu-Jung Lai^2,3,4^, Chia-Yu Chen^4^, Cheng-Han Tsai^4^, Shih-Hsiung Wu^4^ and Yu-Hsuan Tsai^1^

^1^School of Chemistry, Cardiff University, Cardiff, UK

^2^Graduate Institute of Biomedical Sciences, China Medical University, Taichung, Taiwan

^3^Research Center for Cancer Biology, China Medical University, Taichung, Taiwan

^4^Institute of Biological Chemistry, Academia Sinica, Taipei, Taiwan

Correspondence and requests for materials should be addressed to Y.H.T. (email: [tsaiy5@cardiff.ac.uk](mailto:tsaiy5@cardiff.ac.uk))

**Supplementary Information**

**Contents**

[Supplementary Figures 2](#_Toc55051964)

[Supplementary Tables 23](#_Toc55051965)

[Supplementary Methods 24](#_Toc55051966)

[Reference 25](#_Toc55051967)

# Supplementary Figures

**
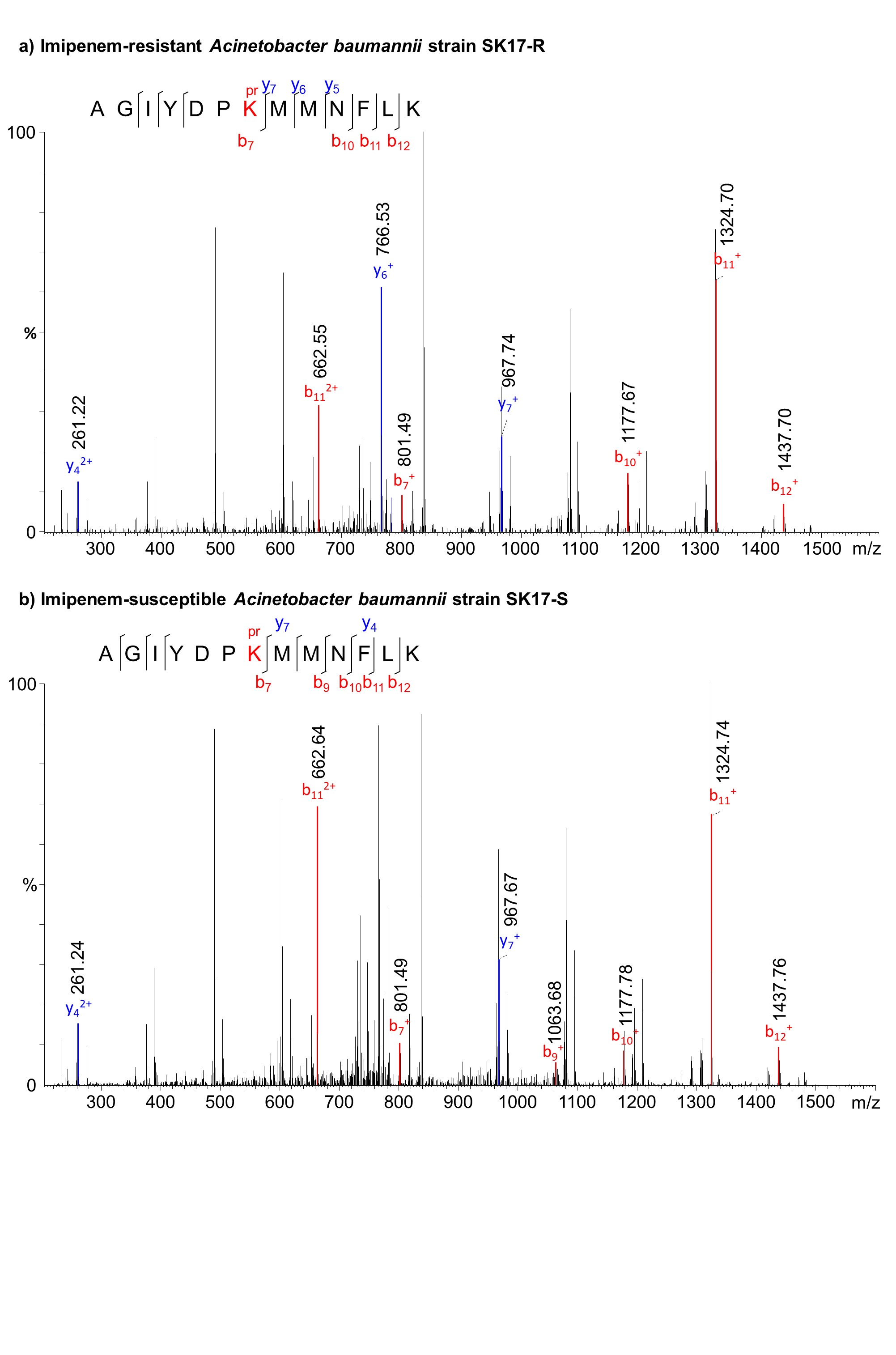
**

**Figure S1.** Tandem mass spectrometry of AGIYDPKprMMNFLK peptide from *Acinetobacter baumannii* a) SK17-R and b) SK17-S.

**
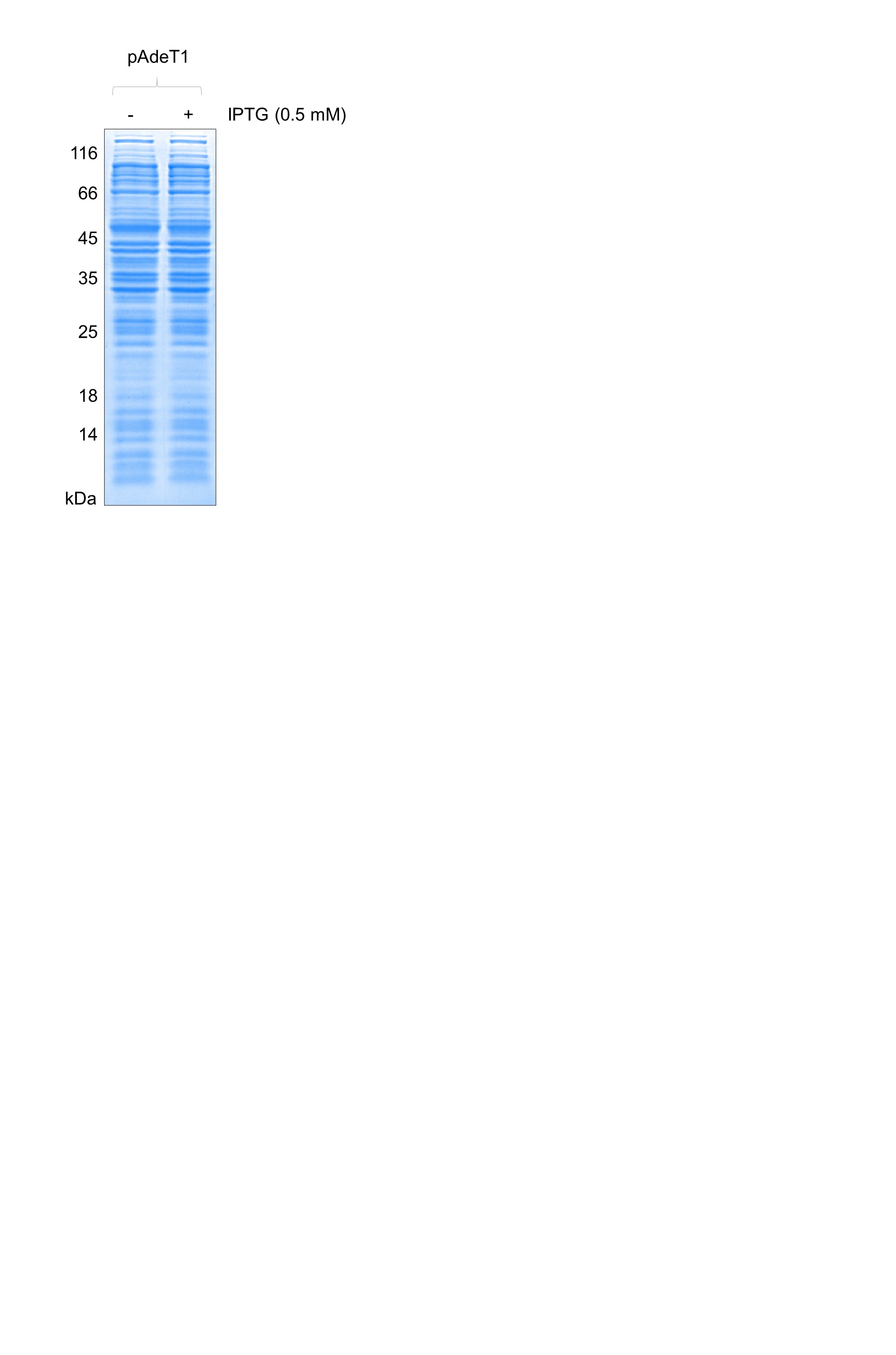
**

**Figure S2.** SDS-PAGE analysis of AdeT1 expression from *E. coli* KAM32 pAdeT1. The pAdeT1 plasmid was constructed as described in the literature ^1^. No band at the expected molecular weight (33.4 kDa) was observed after induction with 0.5 mM IPTG.


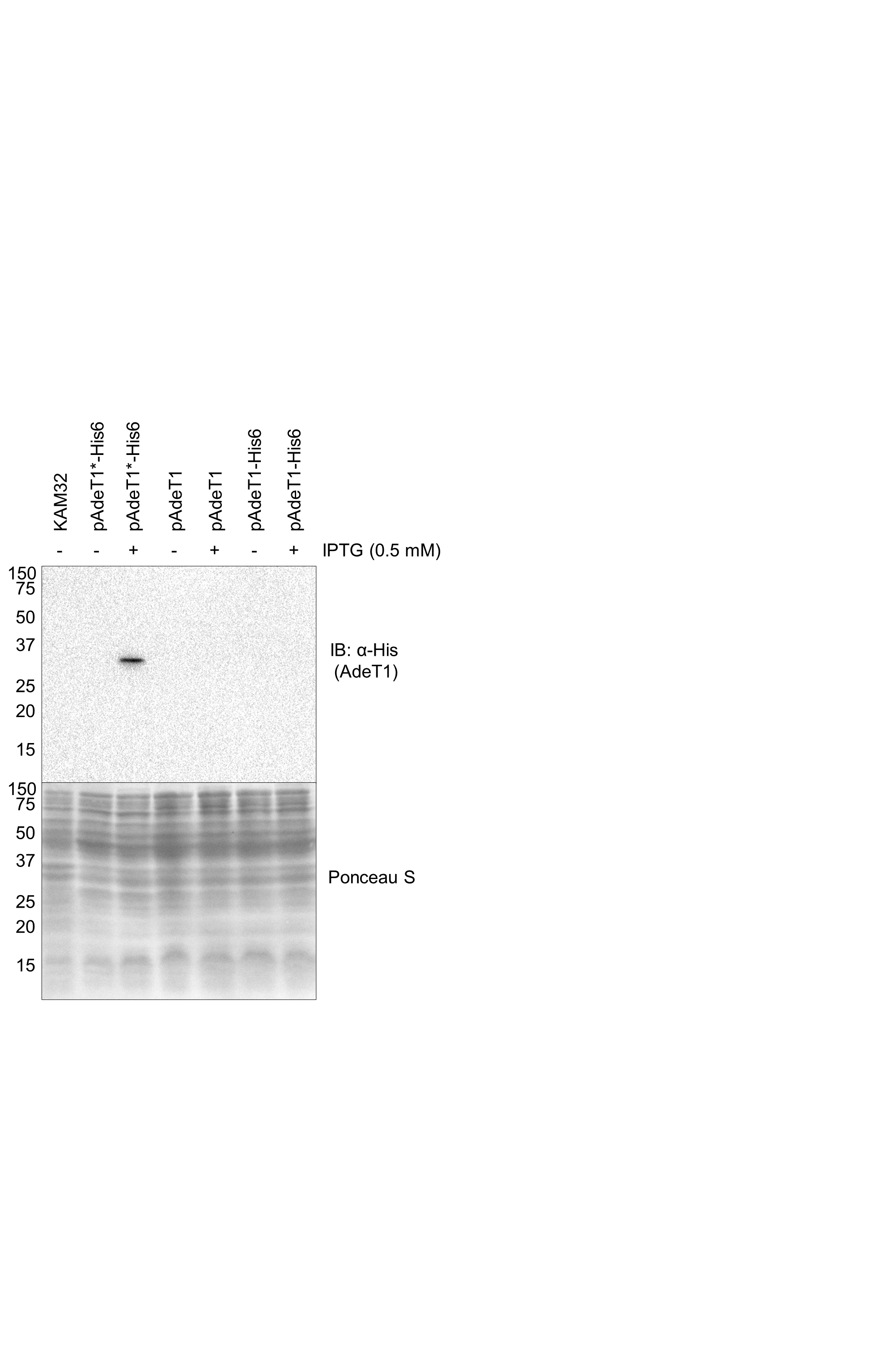


**Figure S3.** Immunoblotting analysis of AdeT1 expression in *E. coli* KAM32 from three plasmids. The pAdeT1 plasmid was constructed as described before^2^, while pAdeT1-His6 contains a 6x Histidine tag on the C terminus of the protein encoding gene. The pAdeT1*-His6 plasmid is a similar construct, with the *adeT1* gene in frame with a start codon and with a 6x Histidine tag on the C terminus. Cells were induced with 0.5 mM IPTG at OD_600_ ~ 0.6. After 2 h at 37 °C, samples were taken for analysis. This figure is the full-length version of the cropped immunoblot from Figure 2.


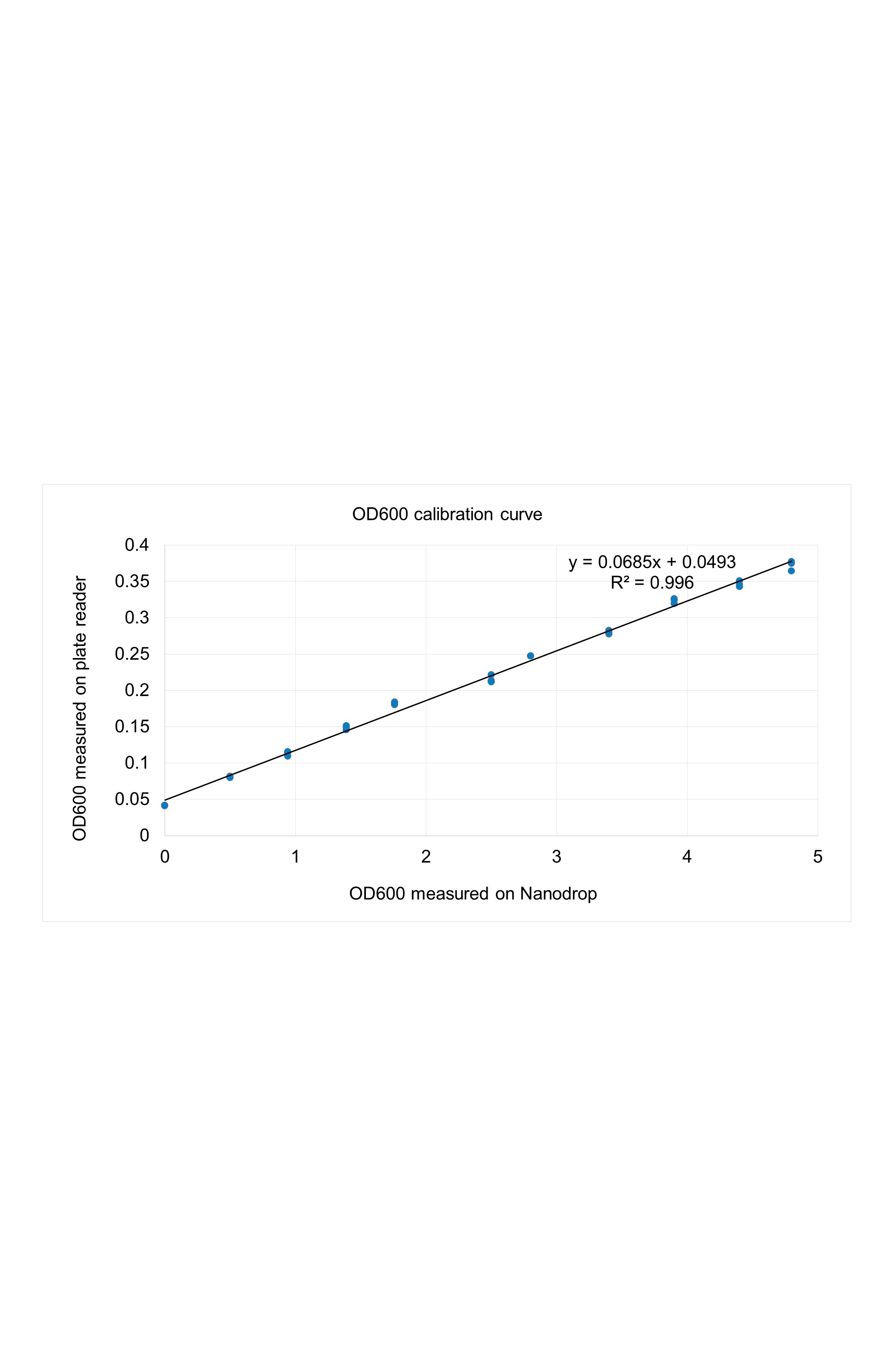


**Figure S4.** OD_600_ calibration curve for comparison of OD_600_ values measured on a Victor X plate reader to those measured by a NanoDrop One. *E. coli* KAM32 cells were cultured overnight at 37 °C, 180 rpm. The culture was then diluted in fresh media to a variety of cell densities. Absorbance at 600 nm of 1 mL of each dilution was measured in a cuvette using a NanoDrop One (Thermo Fisher). The same sample was then dispensed in triplicate into a flat-bottom 96 well plate, with 100 µL per well and absorbance at 600 nm measured by a Victor X (Perkin Elmer) plate reader (CW-lamp OG590, filter B7).


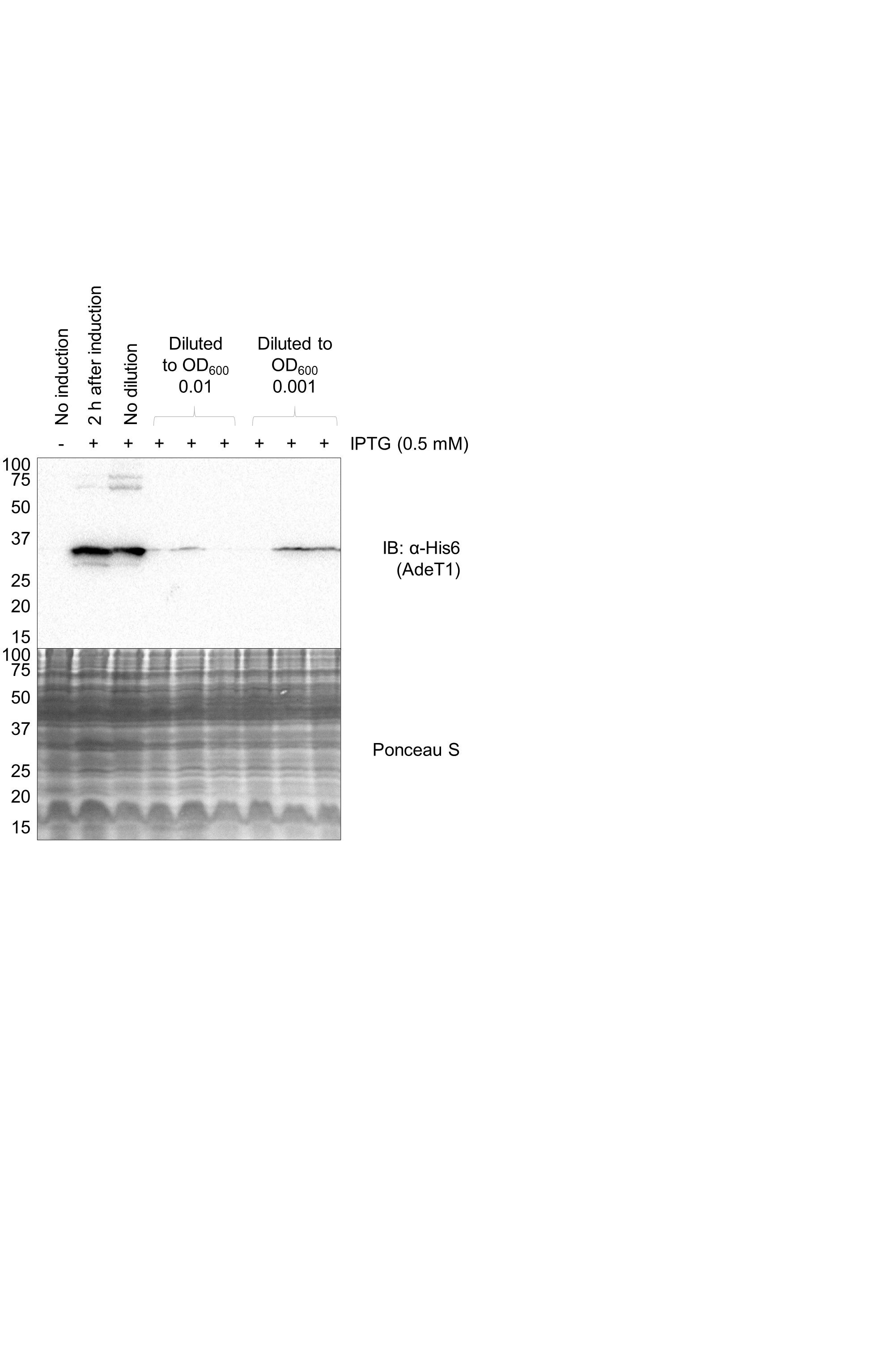


**Figure S5.** AdeT1 production was not always detectable after dilution of the induced culture to low optical density and subsequent overnight incubation in the presence of IPTG. *E. coli* KAM32 carrying pAdeT1*-His6 were induced with 0.5 mM IPTG at OD_600_ ~ 0.6. After 2 h, the induced culture was diluted to either OD_600_ 0.01 or 0.001 in fresh media containing 0.5 mM IPTG and 100 μg/mL ampicillin (for plasmid selection). Three technical replicates were prepared for each dilution. This figure is the full-length version of the cropped immunoblot in Figure 3.


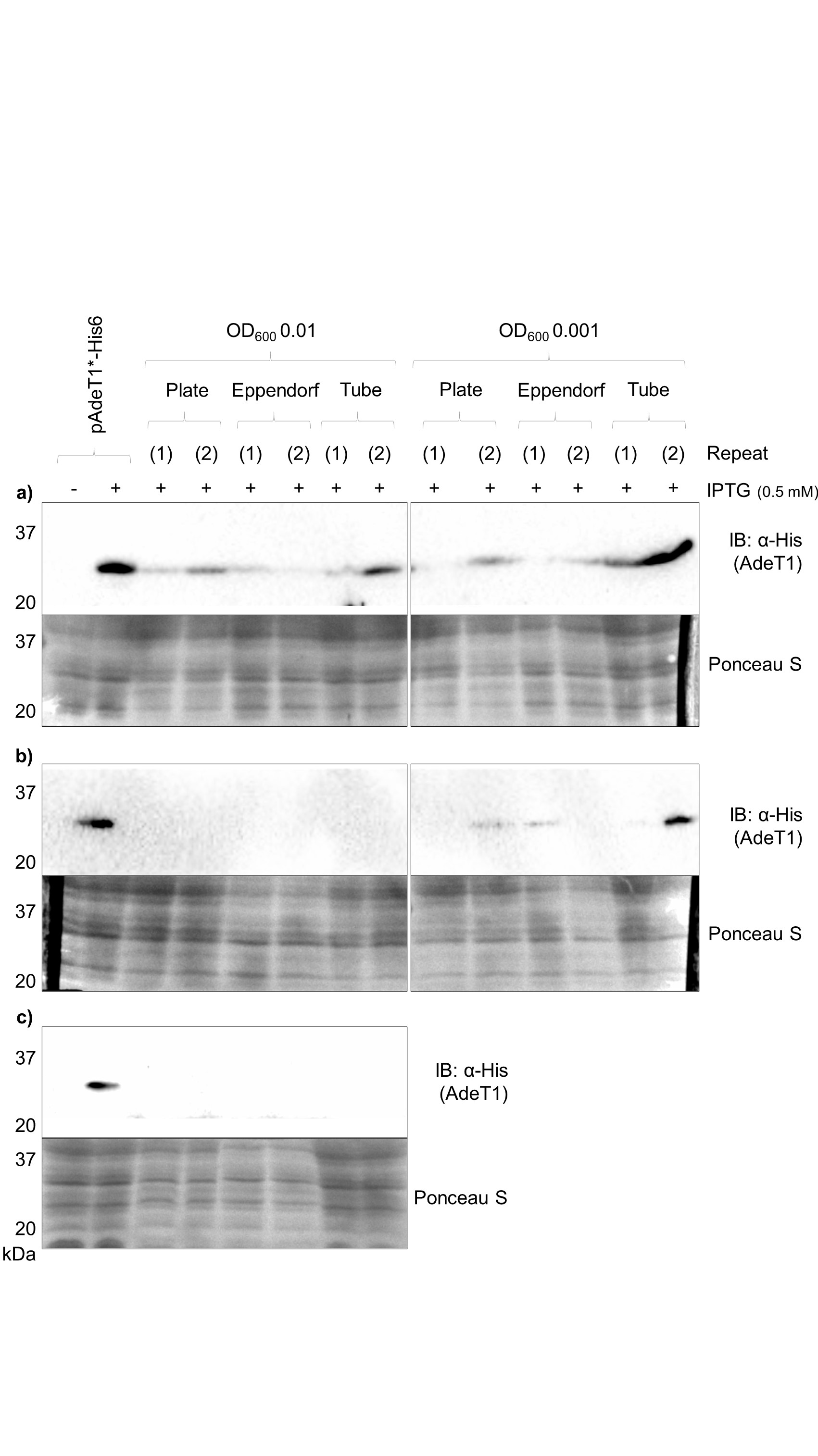


**Figure S6.** Western Blot analysis of expression of *E. coli* KAM32 pAdeT1*-His6 when diluted to low optical densities. Protein expression was detected through the 6x Histidine tag on the C terminus. Cultures were grown until OD_600_ 0.6 before induction with 0.5 mM IPTG. 2 hours post-induction, samples of the induced culture (+) and a no IPTG control (-) were taken and culture diluted to OD_600_ 0.01 or 0.001 in a 96-well plate, Eppendorf or falcon tube. Three biological repeats, a), b), and c) were conducted on consecutive days. On the third day (c), no bacterial growth was observed when the culture was diluted to OD_600_ 0.001.


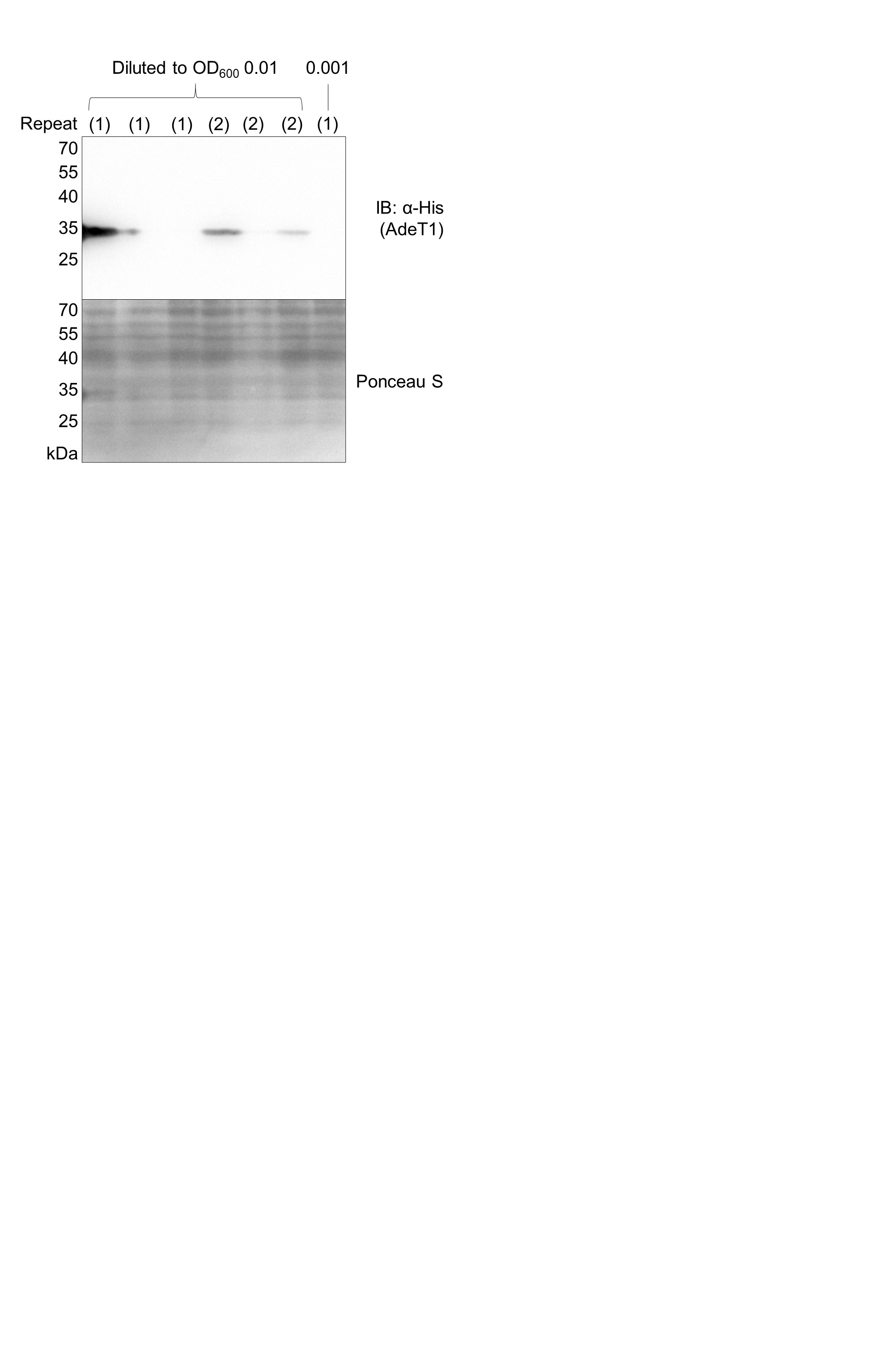


**Figure S7.** Western blot analysis of BL21(DE3) pET28a AdeT1*-His6 before and after induction with 0.5 mM IPTG, and after dilution into Mueller Hinton Broth (MHB) with 0.5 mM IPTG to OD_600_ 0.01 and 0.001. Protein was detected through the 6x Histidine tag on the C terminus.


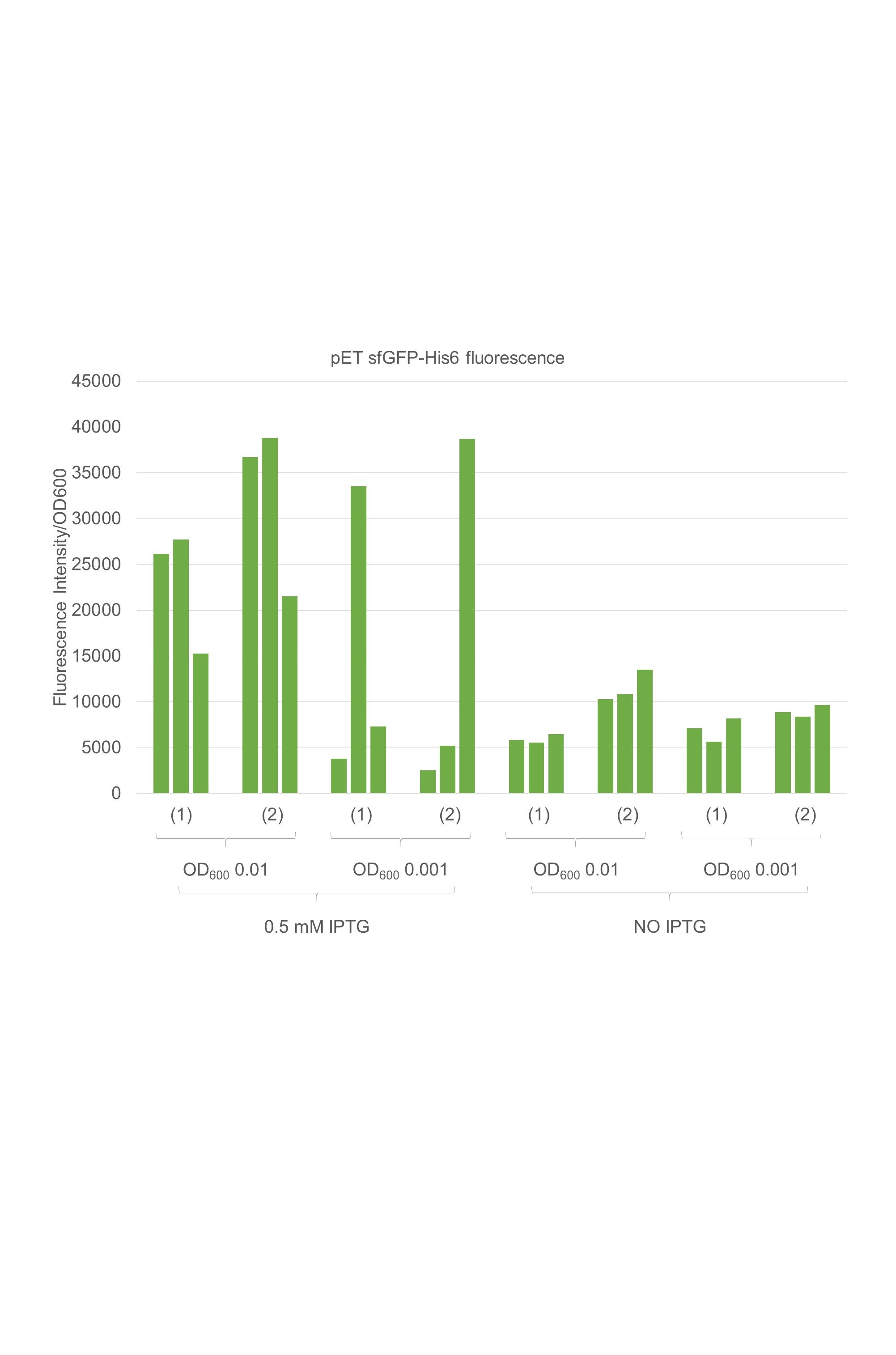


**Figure S8.** Fluorescence intensity of *E. coli* BL21(DE3) cells expressing pET28a sfGFP-His6 when diluted to OD_600_ 0.01 or 0.001 into fresh IPTG-containing media, 1 hour post-induction with IPTG. Cells were then incubated for 18 hours before being pelleted and resuspended in PBS. Fluorescence intensity was measured with a FLUOstar (Optima) plate reader (ex 485 nm, em 520 nm, gain 804) and absorbance at 600 nm was measured with a Victor X (Perkin Elmer) plate reader (CW-lamp OG590, filter B7). For each condition, two independent transformations, (1) and (2), were diluted three times to produce three technical repeats (bars on chart).


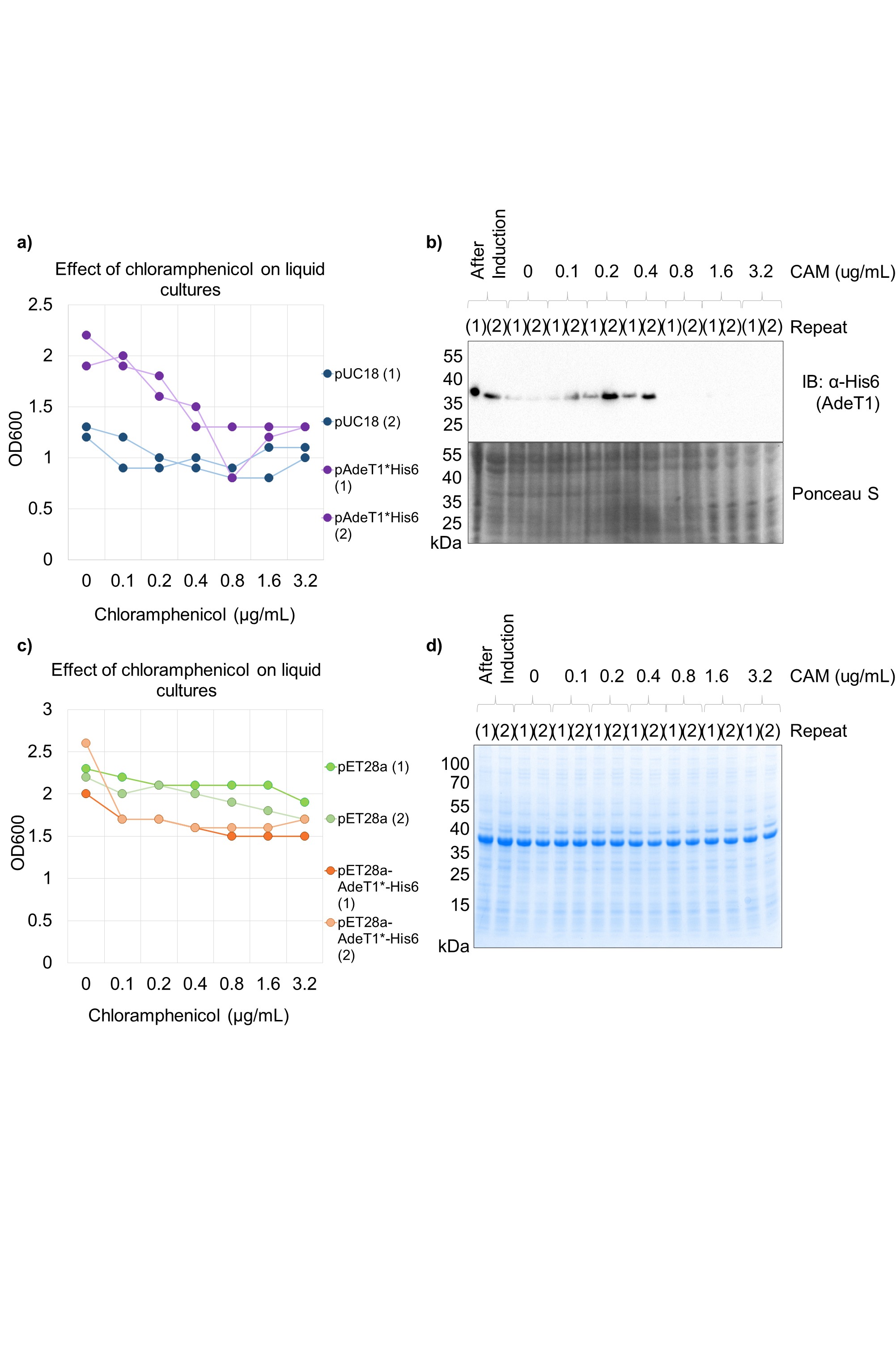


**Figure S9.** Attempts to determine the MIC through direct addition of chloramphenicol to the liquid cultures, without prior dilution. Cells were cultured until OD_600_ 0.6, when induced with 0.5 mM IPTG. 2 hours post-induction, cultures were split evenly and varying amounts of chloramphenicol added before overnight incubation. a) OD_600_ of *E. coli* KAM32 pAdeT1 or pUC18 after overnight incubation with chloramphenicol. b) Western blot analysis of AdeT1 production in *E. coli* KAM32 pAdeT1 cultures after overnight incubation with chloramphenicol. Protein was detected through the 6x Histidine tag on the C terminus. c) OD_600_ of *E. coli* BL21(DE3) pET28a AdeT1*His6 or pET28a after overnight incubation with chloramphenicol. d) SDS-PAGE analysis of AdeT1 production in *E. coli* BL21(DE3) pET28a AdeT1-His6 after overnight incubation with chloramphenicol.


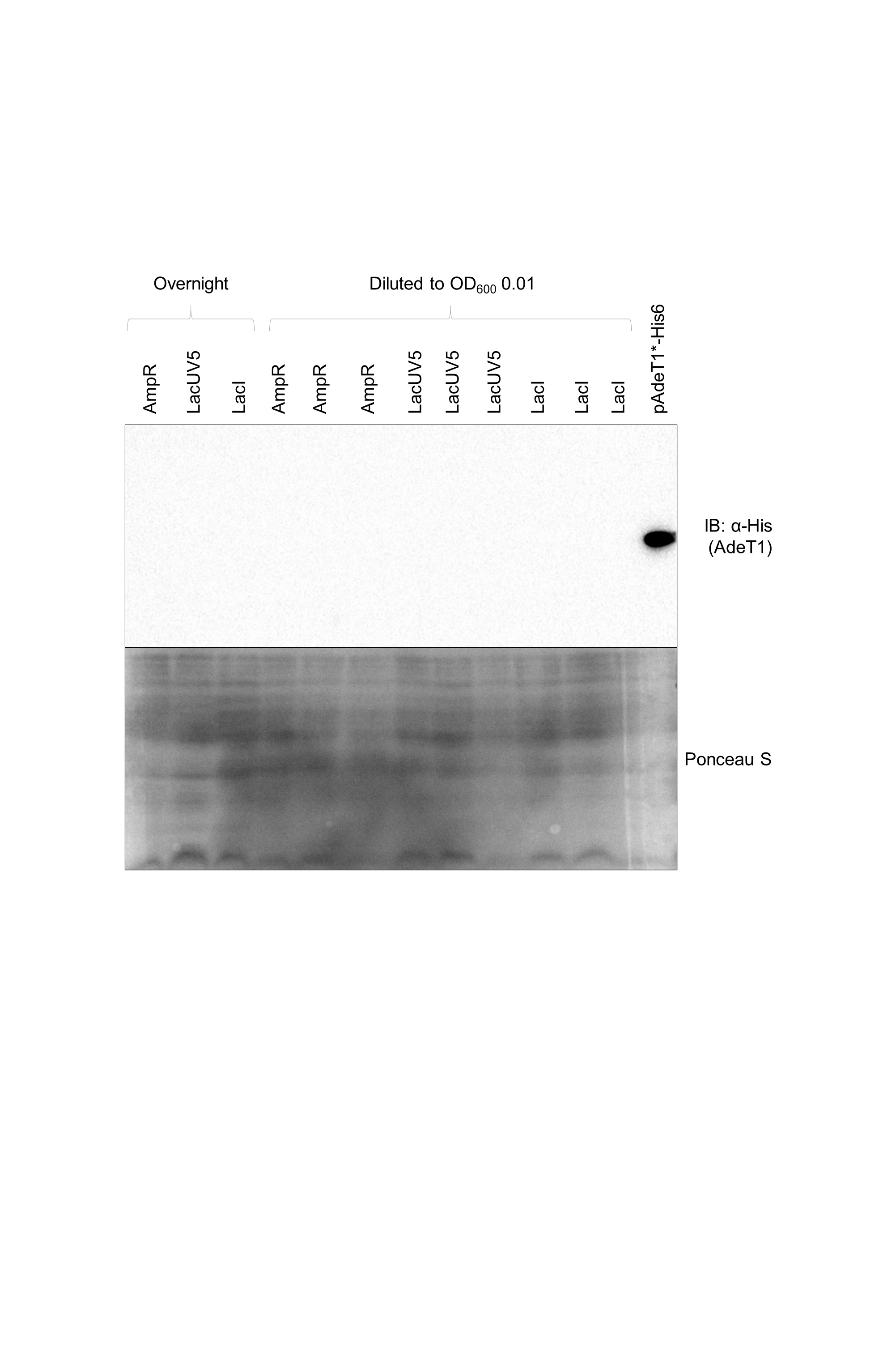


**Figure S10.** Immunoblotting analysis of AdeT1 production under the control of constitutively active promoters. A single colony was cultured overnight in MHB, then diluted to OD_600_ 0.01 with three replicates and cultured overnight. This figure is the full-length version of the cropped immunoblot in Figure 5.


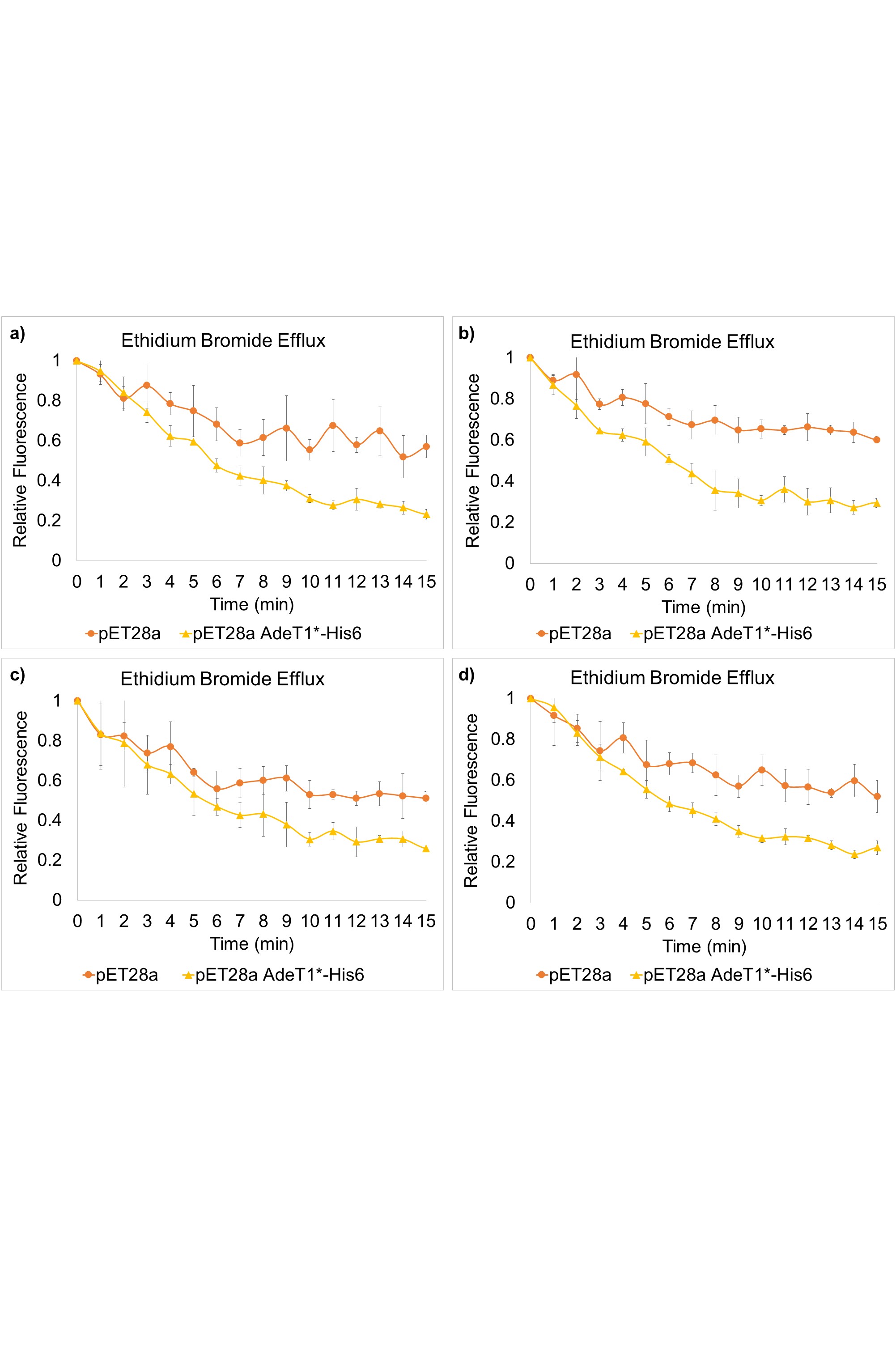


**Figure S11.** Efflux of ethidium bromide (EtBr) from *Escherichia coli* BL21(DE3) cells expressing pET28a or pET28a AdeT1*-His6. Each datapoint represents an average of three technical replica, standard deviation is annotated for each average. Four independent biological repeats (a, b, c, and d) are shown..

**
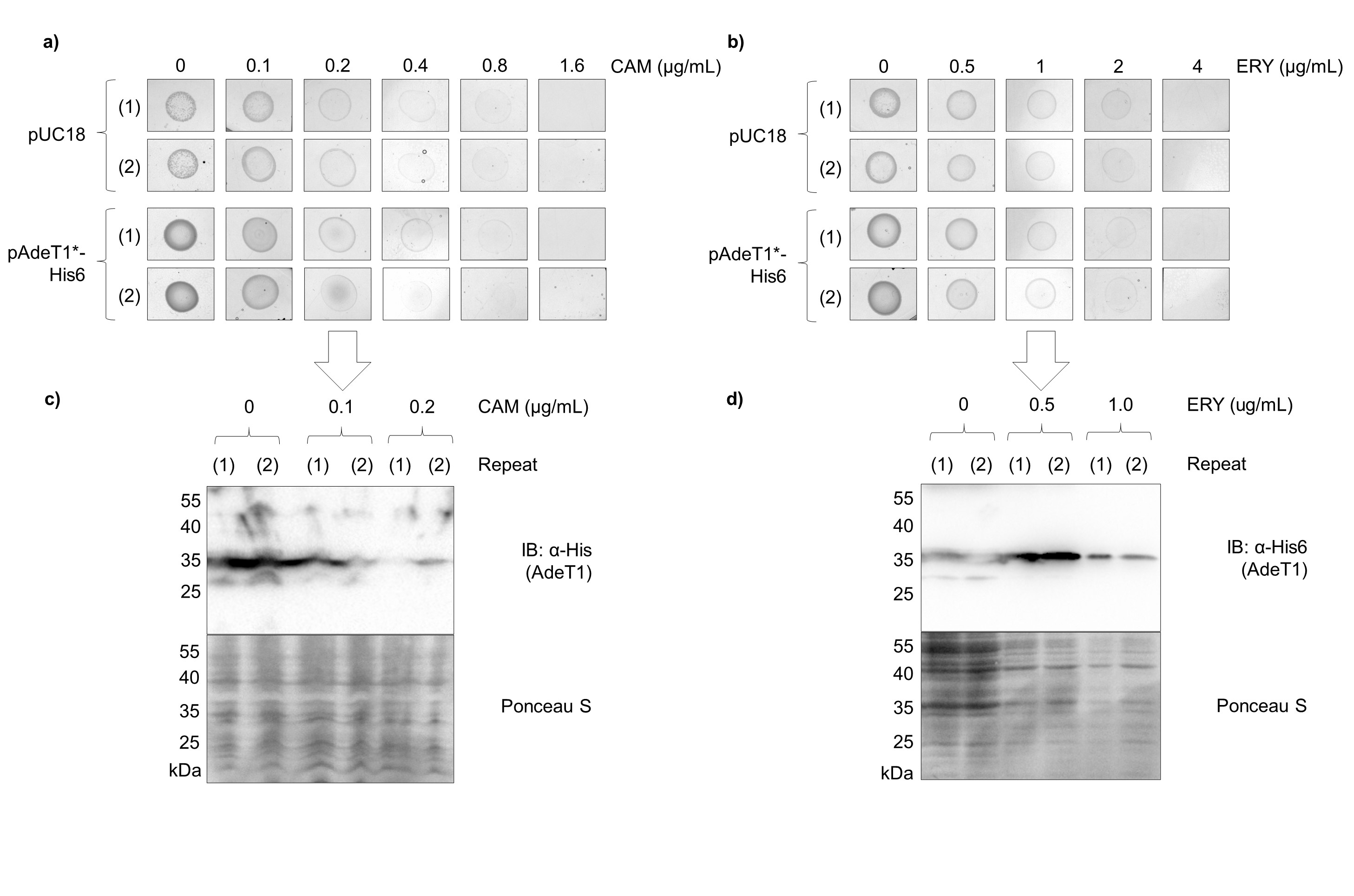
**

**Figure S12.** a) Chloramphenicol (CAM) susceptibility and b) erythromycin (ERY) susceptibility of *E. coli* KAM32 cells carrying either pUC18 or pAdeT1*-His6. Cells were propagated until OD_600_ 0.6, when induced with 0.5 mM IPTG. 2 h after induction, OD_600_ of cultures were normalised to OD_600_ 4.0 and 20 µL dropped on LB agar plates containing plasmid selection antibiotics, 0.5 mM IPTG, and varying concentrations of chloramphenicol or erythromycin. Data of two biological replicates/independent transformations (1 & 2) are shown; c) and d) Colonies of *E. coli* KAM32 carrying pAdeT1*-His6 from a) or b) were collected and analysed by immunoblotting to confirm protein expression. Protein was detected through the 6x His tag at the C terminus. This figure corresponds to Figure 6 and contains the full-length versions of the cropped immunoblots featured there.

**
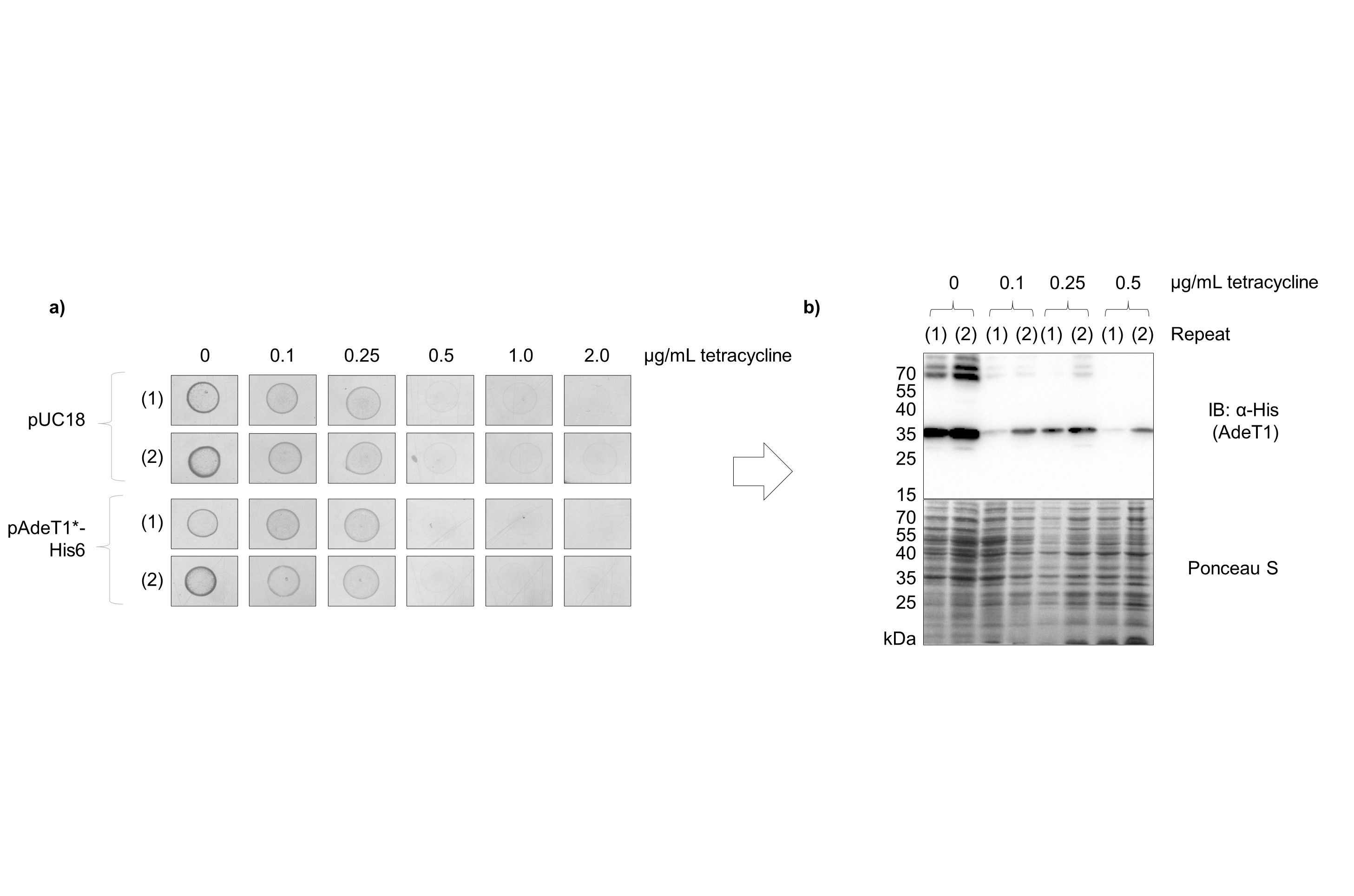
 Figure S13.** a) tetracycline susceptibility of *E. coli* KAM32 cells carrying either pUC18 or pAdeT1*-His6. Cells were propagated until OD_600_ 0.6, when induced with 0.5 mM IPTG. 2 h after induction, OD_600_ of cultures were normalised to OD_600_ 4.0 and 20 µL dropped on LB agar plates containing plasmid selection antibiotics, 0.5 mM IPTG, and varying concentrations of tetracycline. Data of two biological replicates/independent transformations (1 & 2) are shown; b) Colonies of *E. coli* KAM32 carrying pAdeT1*-His6 from a) were collected and analysed by immunoblotting to confirm protein expression. Protein was detected through the 6x His tag at the C terminus. This figure corresponds to Figure 6 and contains the full-length versions of the cropped immunoblots featured there.

**
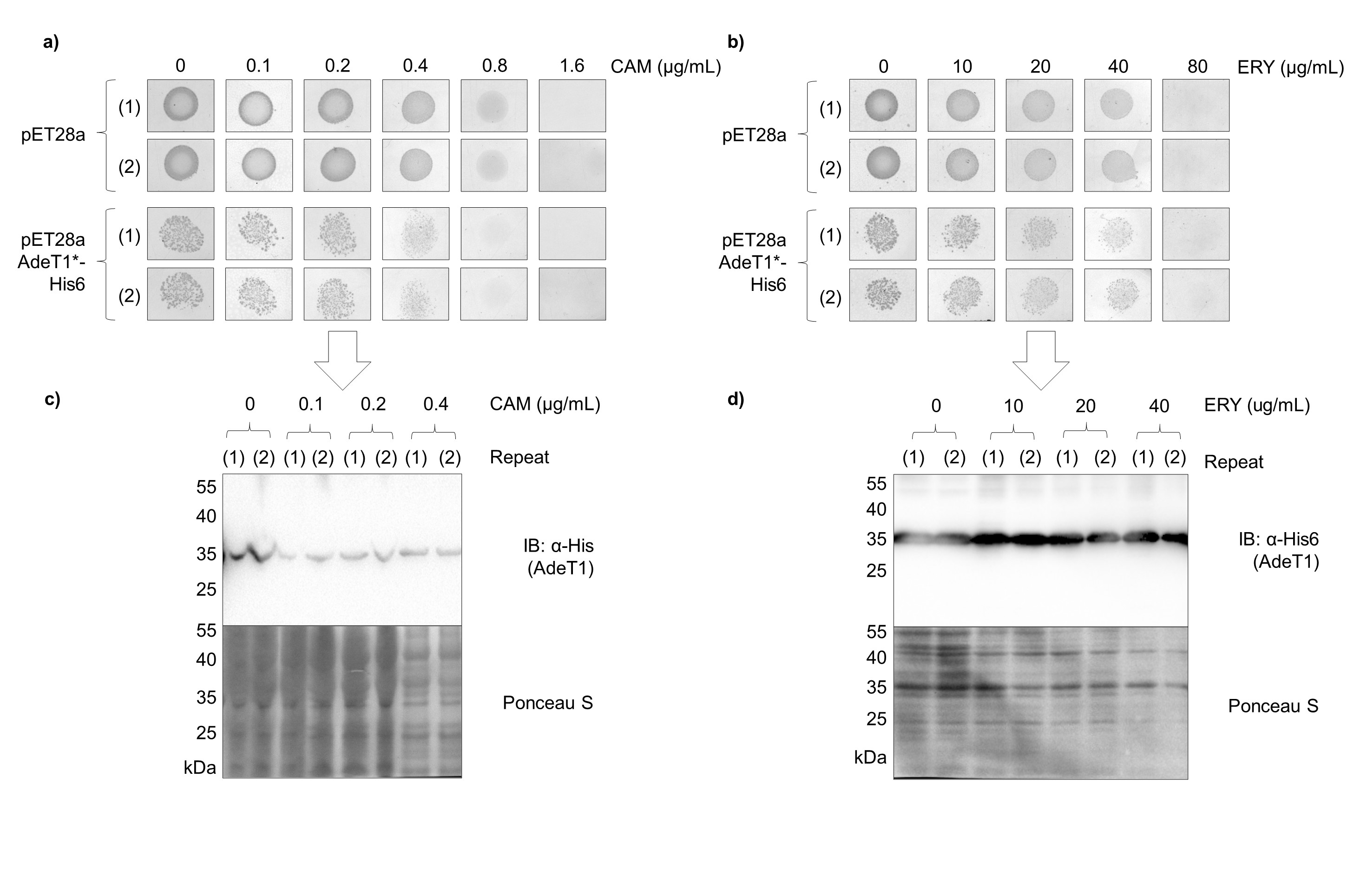
 Figure S14.** a) Chloramphenicol (CAM) susceptibility and b) erythromycin (ERY) susceptibility of *E. coli* BL21(DE3) carrying either pET28a or pET28a AdeT1*-His6. Cells were propagated until OD_600_ 0.6, when induced with 0.5 mM IPTG. 2 h after induction, OD_600_ of cultures were normalised to OD_600_ 4.0 and 20 µL dropped on LB agar plates containing plasmid selection antibiotics, 0.5 mM IPTG, and varying concentrations of chloramphenicol or erythromycin. Data of two biological replicates/independent transformations (1 & 2) are shown; c) and d) Colonies of *E. coli* BL21(DE3) carrying pET AdeT1*-His6 from a) or b) were collected and analysed by immunoblotting to confirm protein expression. Protein was detected through the 6x His tag at the C terminus.


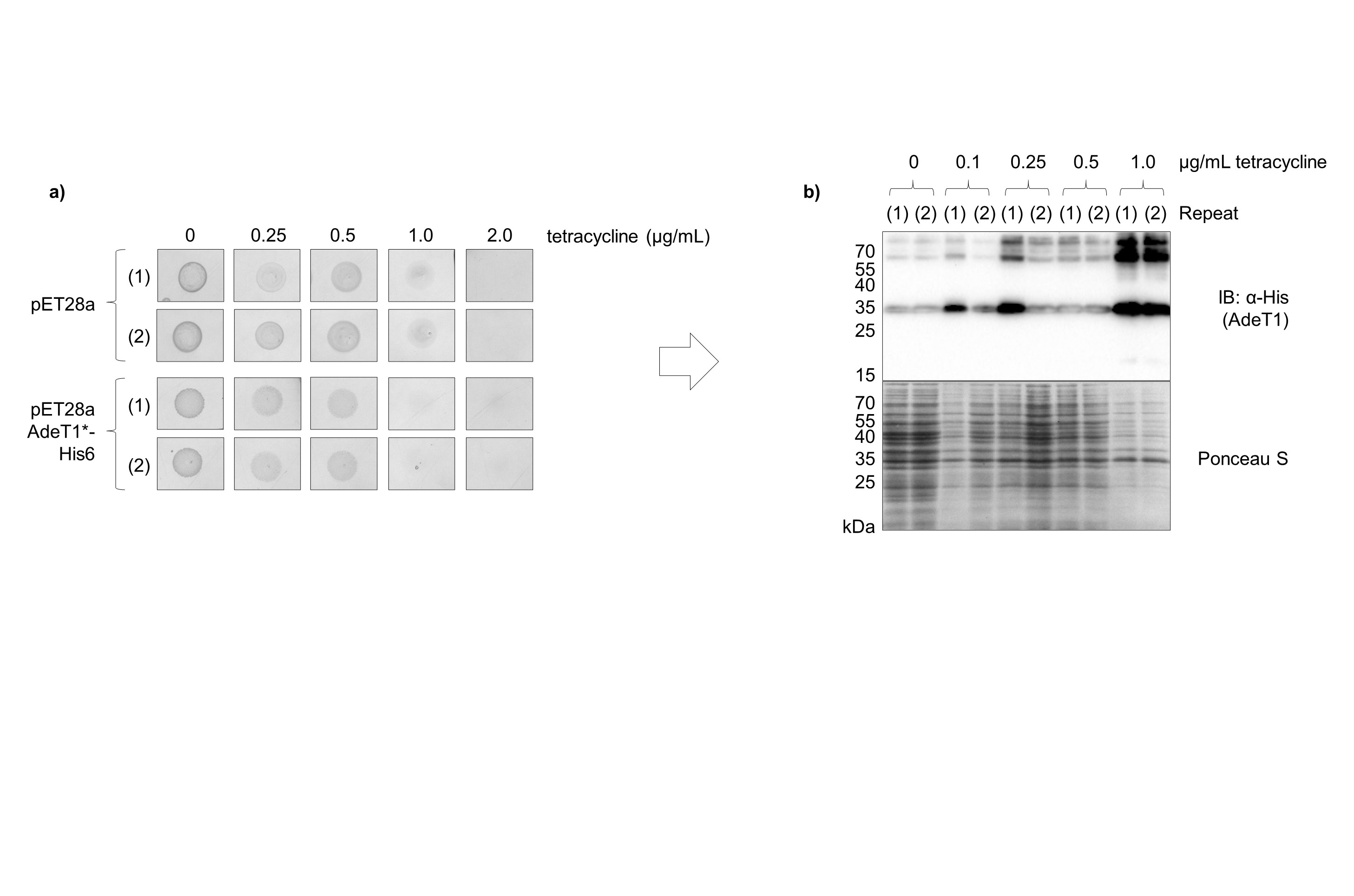


**Figure S15.** a) Tetracycine (TET) susceptibility of *E. coli* BL21(DE3) carrying either pET28a or pET28a AdeT1*-His6. Cells were propagated until OD_600_ 0.6, when induced with 0.5 mM IPTG. 2 h after induction, OD_600_ of cultures were normalised to OD_600_ 4.0 and 20 µL dropped on LB agar plates containing plasmid selection antibiotics, 0.5 mM IPTG, and varying concentrations of tetracycline. Data of two biological replicates/independent transformations (1 & 2) are shown. b) Colonies of *E. coli* BL21(DE3) carrying pET AdeT1*-His6 from a) were collected and analysed by immunoblotting to confirm protein expression. Protein was detected through the 6x His tag at the C terminus.


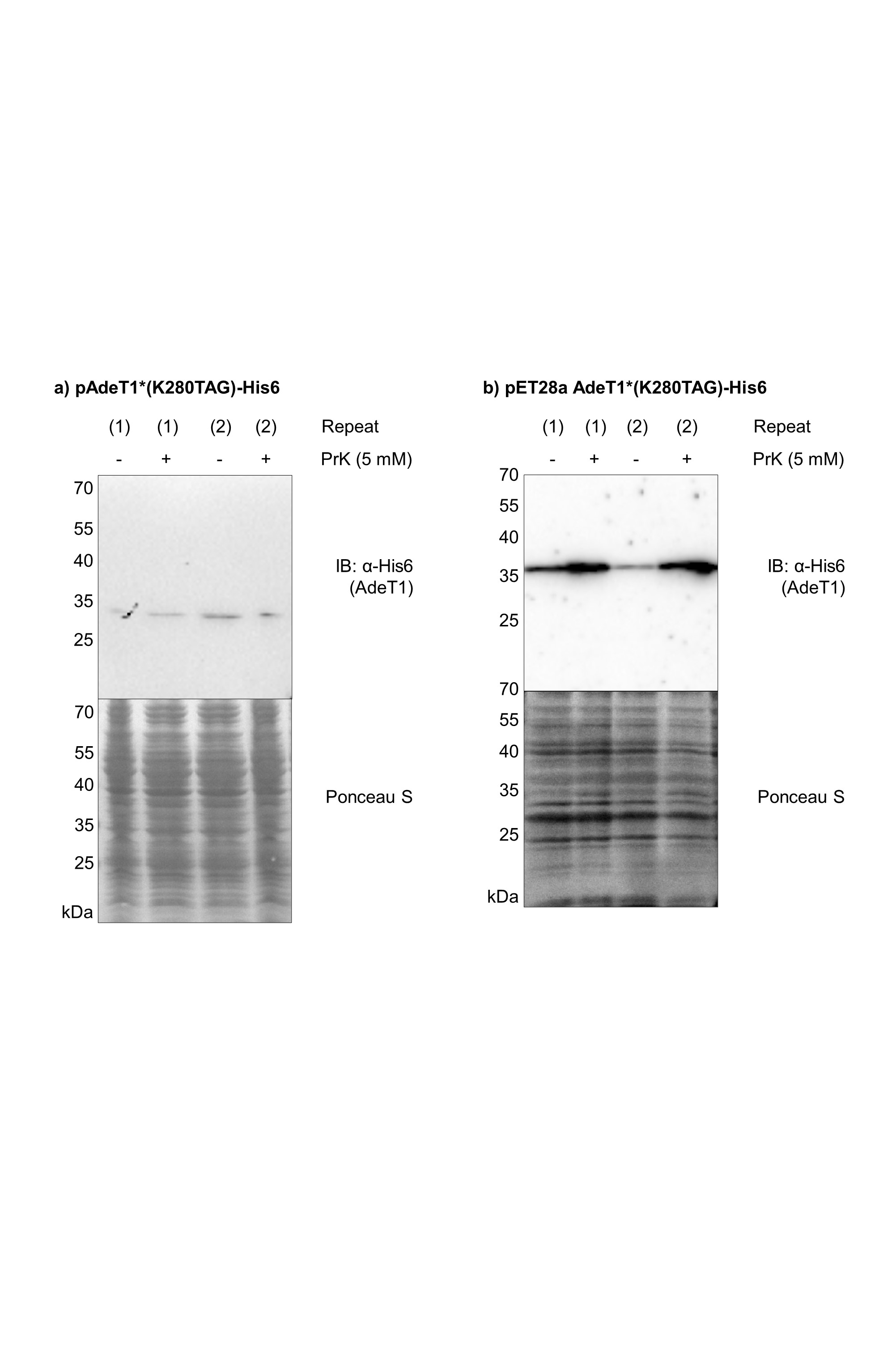


**Figure S16.** Propionyl-lysine (PrK) incorporation into AdeT1 protein. *E. coli* KAM32 cells (a) or *E. coli* BL21(DE3) cells (b) were co-transformed with either pAdeT1*(K280TAG)-His6 (a) or pET28a AdeT1*(K280TAG)-His6 (b) and pAcKST. Cells were grown until OD_600_ 0.6 before induction with 0.5 mM IPTG. Cultures were then split into 2 halves. 5 mM PrK was added to one half, while the other culture was kept as a control. Cells were incubated for 2 hours at 37 °C before samples were taken and analysed by immunoblotting to verify protein expression. Protein was detected through the 6x Histidine tag on the C terminus. Data of two biological repeats/independent transformations (1 & 2) are shown.

**
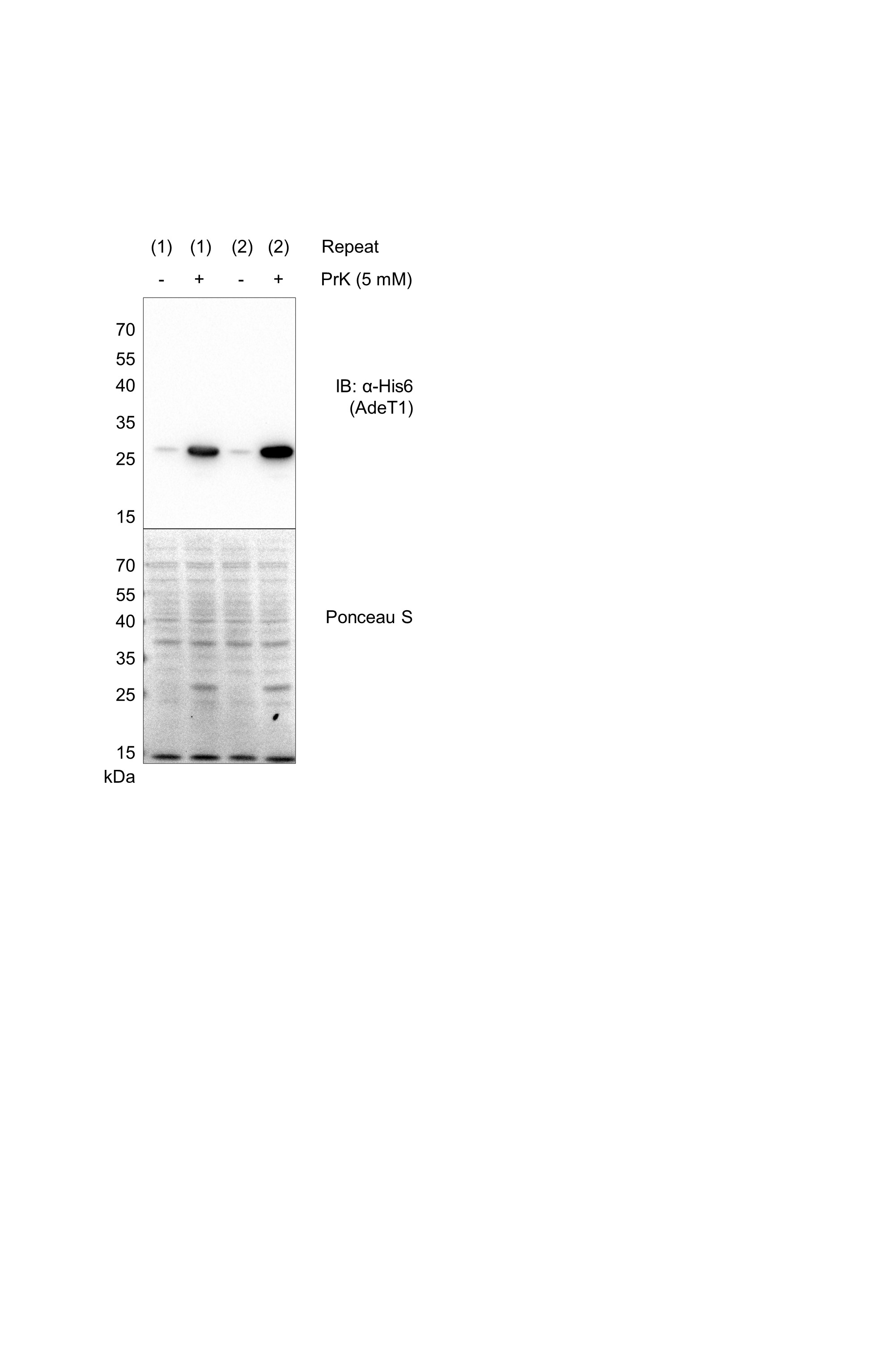
**

**Figure S17.** Propionyl-lysine (PrK) incorporation into sfGFP protein. *E. coli* BL21(DE3) cells were co-transformed with pET28a sfGFP(K150TAG)-His6 and pAcKST. Cells were grown until OD_600_ 0.6 before induction with 0.5 mM IPTG. Cultures were then split into 2 halves. 5 mM PrK was added to one half, while the other culture was kept as a control. Cells were incubated for 2 hours at 37 °C before samples were taken and analysed by immunoblotting to verify protein expression. Protein was detected through the 6x Histidine tag on the C terminus. Data of two biological repeats/independent transformations (1 & 2) are shown.

**
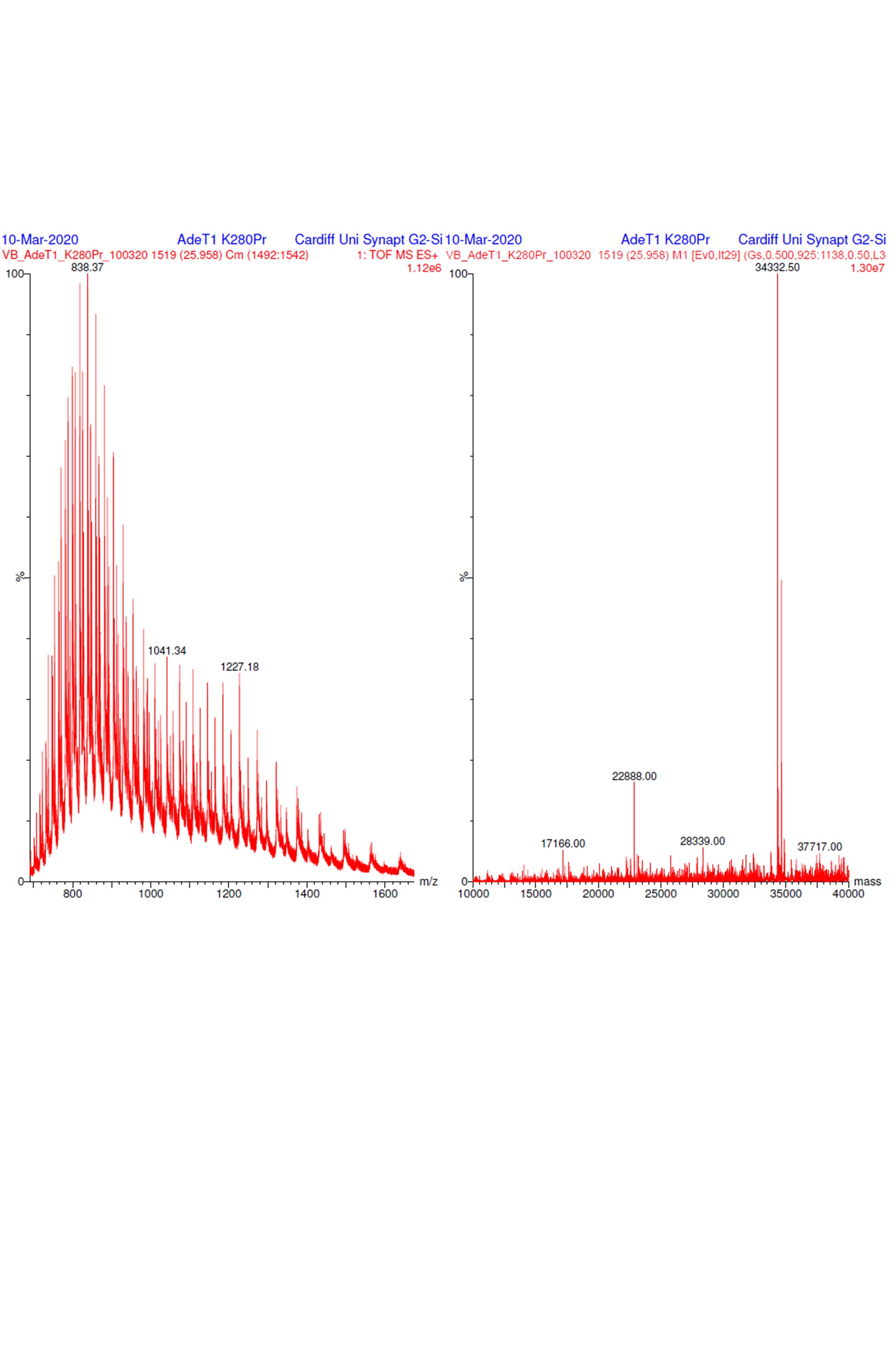
**

**Figure S18**. Mass spectrometry analysis of AdeT1 protein with propionyl-lysine incorporated into amino acid position 280. *E. coli* BL21(DE3) cells expressing pET28a AdeT1(K280TAG)-His6 were cultured to OD_600_ 0.6, before induction with 0.5 mM IPTG and supplementation with 5 mM PrK. Cells were incubated for 18 hours at 20 °C before being harvested and purified by nickel affinity chromatography. The expected average mass is 34334.53 Da. A peak of 34332.50 Da can be observed. Samples were analysed using a SYNAPT G2-Si (Waters) mass spectrometer.


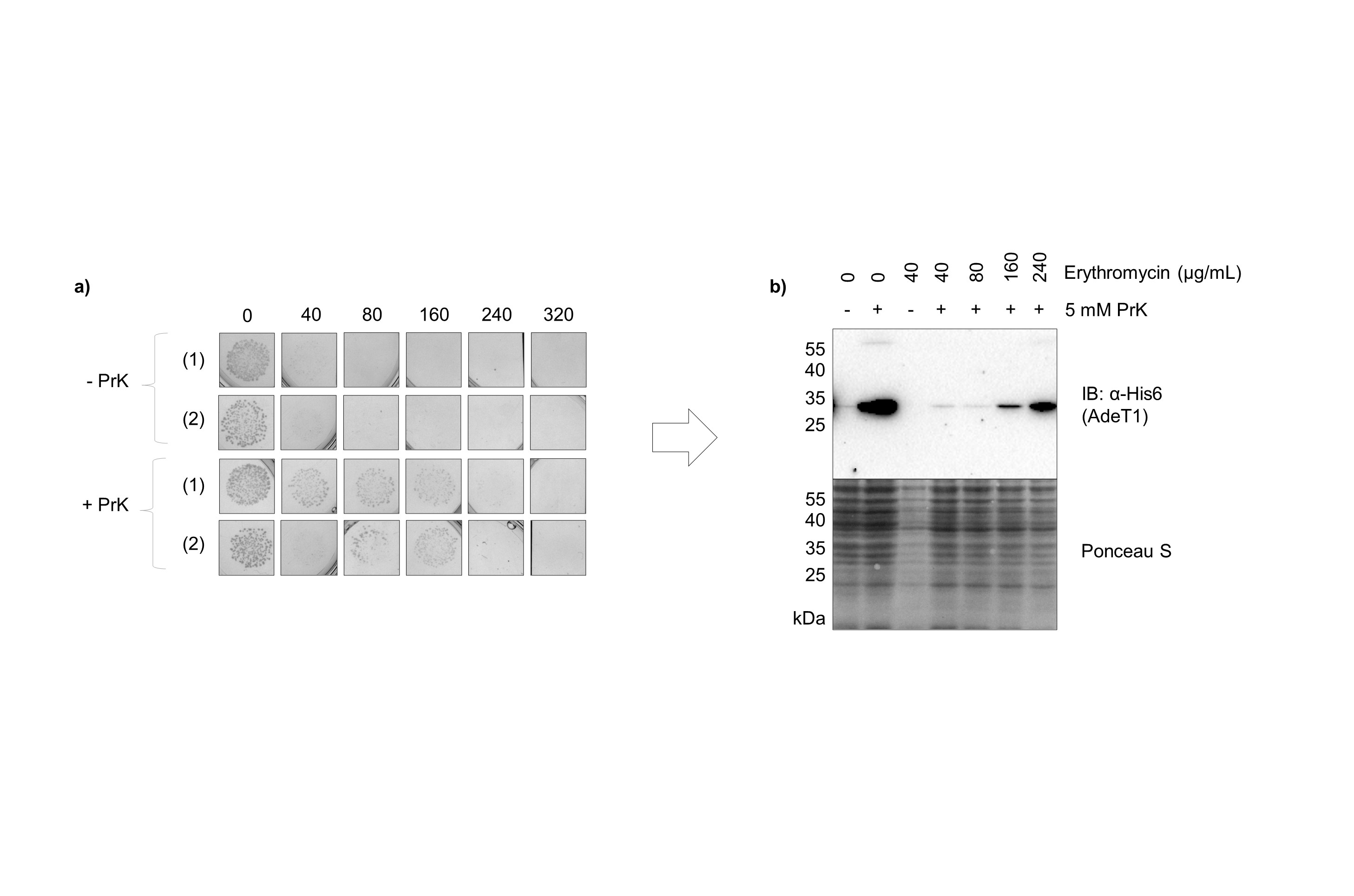


**Figure S19.** a) Erythromycin susceptibility of *E. coli* BL21(DE3) carrying pET28a AdeT1*(K280TAG)-His6 and pAcKST. Cells were propagated until OD_600_ 0.6, when induced with 0.5 mM IPTG. The culture was then split in two, and one supplemented with propionyl-lysine (+ PrK) while the other left as a control (- PrK). 2 h after induction, OD_600_ of cultures were normalised to OD_600_ 4.0 and 20 µL dropped on LB agar plates containing plasmid selection antibiotics, 0.5 mM IPTG, and varying concentrations of erythromycin. Data of two biological replicates/independent transformations (1 & 2) are shown. b) Colonies of *E. coli* BL21(DE3) from a) were collected and analysed by immunoblotting to confirm protein expression. Protein was detected through the 6x His tag at the C terminus.


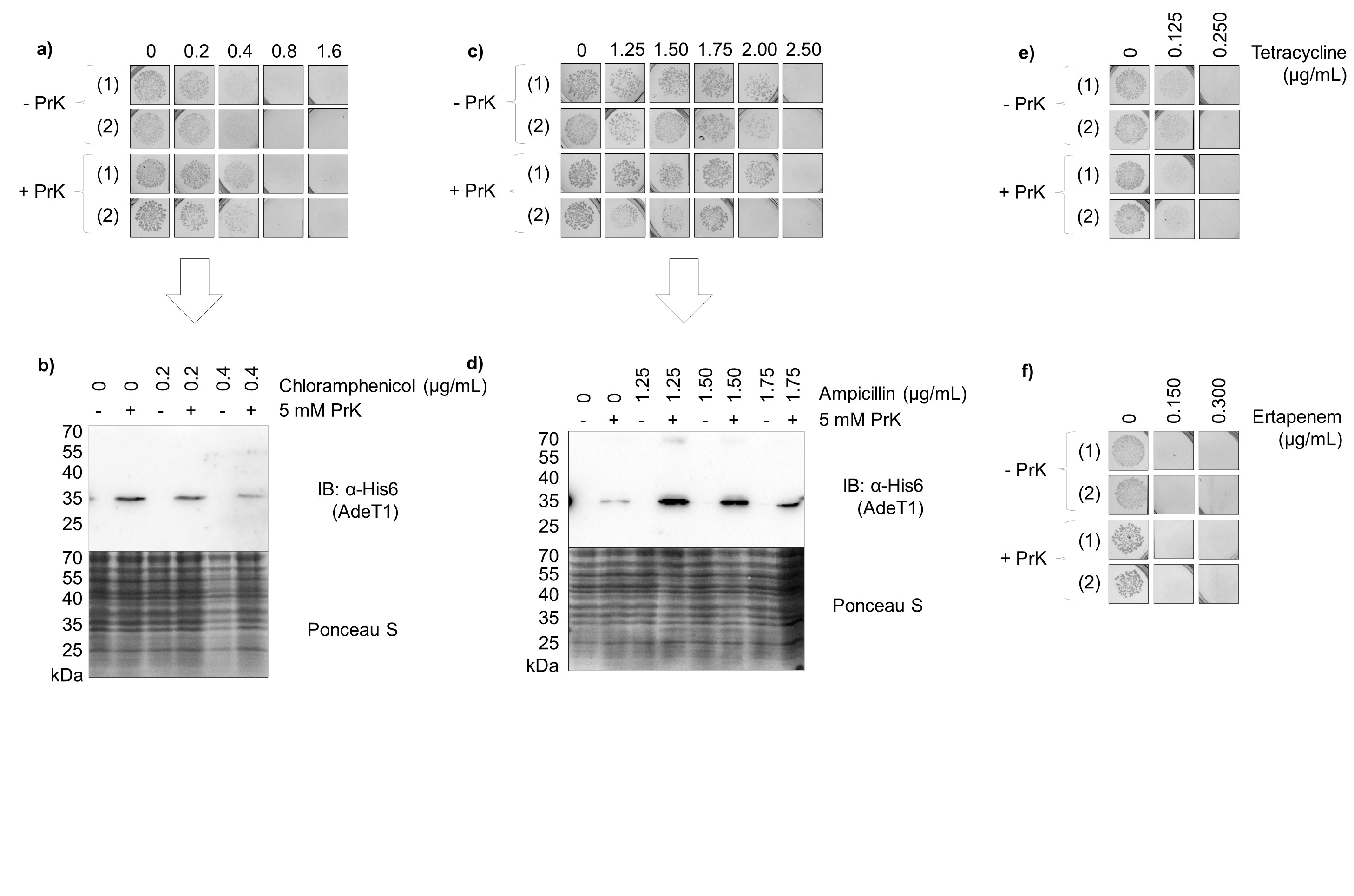


**Figure S20.** a) Chloramphenicol; c) ampicillin; e) tetracycline and f) ertapenem susceptibility of *E. coli* BL21(DE3) carrying pET28a AdeT1*(K280TAG)-His6 and pAcKST. Cells were propagated until OD_600_ 0.6, when induced with 0.5 mM IPTG. The culture was then split in two, and one supplemented with propionyl-lysine (+ PrK) while the other left as a control (- PrK). 2 h after induction, OD_600_ of cultures were normalised to OD_600_ 4.0 and 20 µL dropped on LB agar plates containing plasmid selection antibiotics, 0.5 mM IPTG, and varying concentrations of erythromycin. Data of two biological replicates/independent transformations (1 & 2) are shown. b) and d) Colonies of *E. coli* BL21(DE3) from a) and c) respectively were collected and analysed by immunoblotting to confirm protein expression. Protein was detected through the 6x His tag at the C terminus.

# Supplementary Tables

|  | Chloramphenicol (µg/mL) | | | | | | | | |
| --- | --- | --- | --- | --- | --- | --- | --- | --- | --- |
|  | 6.4 | 3.2 | 1.6 | 0.8 | 0.4 | 0.2 | 0.1 | 0.05 | 0 |
| pUC18 | 0.040  ± 0.002 | 0.041  ± 0.001 | 0.040  ± 0.002 | 0.041  ± 0.003 | 0.041  ± 0.002 | 0.046  ± 0.012 | 0.092  ± 0.024 | 0.180  ± 0.031 | 0.241  ± 0.085 |
| pAdeT1* | 0.039  ± 0.002 | 0.040  ± 0.001 | 0.041  ± 0.002 | 0.041  ± 0.003 | 0.044  ± 0.004 | 0.042  ± 0.003 | 0.075  ± 0.030 | 0.143  ± 0.040 | 0.202  ± 0.034 |
| pAdeT1*-His6 | 0.040  ± 0.002 | 0.042  ± 0.001 | 0.040  ± 0.001 | 0.042± 0.004 | 0.041  ± 0.004 | 0.042  ± 0.003 | 0.065  ± 0.034 | 0.160  ± 0.031 | 0.225  ± 0.047 |

**Table S1.** Average OD_600_ measurements of chloramphenicol MIC test cultures as measured by a Victor X (Perkin Elmer) plate reader. *E. coli* KAM32 cells carrying either pUC18, pAdeT1* or pAdeT1*-His6 underwent MIC testing in microdilution plates according to the CLSI protocol.^3^ Each data value is the average of 6 biological replica, the standard deviation is provided.

|  | Erythromycin (µg/mL) | | | | | | | | |
| --- | --- | --- | --- | --- | --- | --- | --- | --- | --- |
|  | 8 | 4 | 2 | 1 | 0.5 | 0.25 | 0.125 | 0.0625 | 0 |
| pUC18 | 0.038  ± 0.002 | 0.040  ± 0.001 | 0.040  ± 0.001 | 0.040  ± 0.001 | 0.068  ± 0.029 | 0.177  ± 0.038 | 0.204  ± 0.025 | 0.237  ± 0.022 | 0.262  ± 0.033 |
| pAdeT1* | 0.039  ± 0.003 | 0.040  ± 0.002 | 0.041  ± 0.003 | 0.045  ± 0.014 | 0.052  ± 0.019 | 0.158  ± 0.039 | 0.165  ± 0.030 | 0.197  ± 0.019 | 0.227  ± 0.036 |
| pAdeT1*-His6 | 0.039  ± 0.001 | 0.041  ± 0.001 | 0.042  ± 0.002 | 0.041  ± 0.002 | 0.050  ± 0.015 | 0.183  ± 0.038 | 0.232  ± 0.083 | 0.249  ± 0.053 | 0.301  ± 0.020 |

**Table S2.** Average OD_600_ measurements of erythromycin MIC test cultures as measured by a Victor X (Perkin Elmer) plate reader. *E. coli* KAM32 cells carrying either pUC18, pAdeT1* or pAdeT1*-His6 underwent MIC testing in microdilution plates according to the CLSI protocol.^3^ Each data value is the average of 6 biological replica, the standard deviation is provided.

|  | Tetracycline (µg/mL) | | | | | | | | |
| --- | --- | --- | --- | --- | --- | --- | --- | --- | --- |
|  | 8 | 4 | 2 | 1 | 0.5 | 0.25 | 0.125 | 0.0625 | 0 |
| pUC18 | 0.038  ± 0.002 | 0.040  ± 0.001 | 0.039  ± 0.001 | 0.038  ± 0.001 | 0.039  ± 0.002 | 0.039  ± 0.002 | 0.042  ± 0.002 | 0.076  ± 0.023 | 0.249  ± 0.033 |
| pAdeT1* | 0.036  ± 0.003 | 0.040  ± 0.002 | 0.039  ± 0.003 | 0.039  ± 0.003 | 0.039  ± 0.002 | 0.038  ± 0.003 | 0.039  ± 0.002 | 0.086  ± 0.029 | 0.212  ± 0.026 |
| pAdeT1*-His6 | 0.038  ± 0.002 | 0.041  ± 0.002 | 0.041  ± 0.002 | 0.041  ± 0.002 | 0.040  ± 0.002 | 0.040  ± 0.004 | 0.039  ± 0.002 | 0.083  ± 0.022 | 0.242  ± 0.067 |

**Table S3.** Average OD_600_ measurements of tetracycline MIC test cultures as measured by a Victor X (Perkin Elmer) plate reader. *E. coli* KAM32 cells carrying either pUC18, pAdeT1* or pAdeT1*-His6 underwent MIC testing in microdilution plates according to the CLSI protocol.^3^ Each data value is the average of 6 biological replica, the standard deviation is provided.

# Supplementary Methods

**Expression of AdeT1(K280Pr)-His6**

*E. coli* BL21(DE3) were co-transformed with pET28a AdeT1*(K280TAG)-His6 and pAcKST. The transformation mixture was used to inoculate 10 mL terrific broth (TB) media containing 100 µg/mL spectinomycin and 50 µg/mL kanamycin, which was incubated for 18 hours at 180 rpm, 37 °C. The following day, the starter culture was used to inoculate 1 L TB containing 100 µg/mL spectinomycin and 50 µg/mL kanamycin to OD_600_ 0.05. The culture was incubated at 180 rpm, 37 °C until OD_600_ 0.6. At this point, the culture was supplemented with 0.5 mM IPTG and 5 mM propionyl-lysine. The temperature was then reduced to 20 °C and the culture incubated for 18 hours at 180 rpm.

**Purification of AdeT1(K280Pr)-His6**

Cultures were centrifuged at 4000 rpm for 20 minutes at 4 °C. The supernatant was discarded, and pellets resuspended in 10 mL chilled lysis buffer (6 M urea, 0.05 M Tris-HCl pH 8.0, 0.15 M NaCl, 0.01 M imidazole, 1 g/L lysozyme, 100 µM PMSF) per 1 g cell pellet. Cells were sonicated on ice in bursts of 5 s ON and 15 s OFF using a microtip at 39% amplification until lysed. The lysate was then centrifuged at 18,000 rpm, 4 °C, for 20 minutes. The supernatant was collected and filtered through a 0.44 µm syringe filter, then combined with Ni-NTA resin equilibrated in lysis buffer and incubated at 4 °C with gentle agitation for 1-2 hours. The lysate/resin was then poured into a gravity column and the flow through collected. The resin was washed with chilled wash buffer (6 M urea, 0.05 M Tris-HCl pH 8.0, 0.15 M NaCl, 0.02 M imidazole) until no protein could be detected in the flow through by a NanoDrop One (ThermoFisher, #ND-ONE-W) measuring protein A_280_ (1 Abs = 1 mg/mL). The protein was then eluted from the column in 2-4 mL fractions using chilled elution buffer (6 M Urea, 0.05 M Tris-HCl pH 8.0, 0.15 M NaCl, 0.25 M imidazole) and analysed by SDS-PAGE.

**Mass spectrometry analysis**

Samples were analysed using SYNAPT G2-Si Mass Spectrometry (Waters) by Cardiff University School of Chemistry Analytical Services.

# Reference

1. Srinivasan, V. B., Rajamohan, G., Pancholi, P., Marcon, M. & Gebreyes, W. A. Molecular cloning and functional characterization of two novel membrane fusion proteins in conferring antimicrobial resistance in Acinetobacter baumannii. *J. Antimicrob. Chemother.* **66**, 499-504 (2011).

2. Srinivasan, V. B., Gebreyes, W. A., Rajamohan, G., Pancholi, P. & Marcon, M. Molecular cloning and functional characterization of two novel membrane fusion proteins in conferring antimicrobial resistance in *Acinetobacter baumannii*. *J. Antimicrob. Chemother.* **66**, 499-504 (2011).

3. CLSI. *Methods for dilution antimicrobial susceptibility tests for bacteria that grow aerobically; approved standard - Ninth edition*. CLSI document M07-A9. Wayne, Pennsylvania, Clinical and Laboratory Standards Institute; 2012
